# Dysregulation of Farnesoid X Receptor on Neutrophil Homeostasis Exacerbates Intestinal Inflammation via the mTORC1‐Glycolysis Signaling Pathway

**DOI:** 10.1002/mco2.70637

**Published:** 2026-02-08

**Authors:** Dengfeng Kang, Ai Li, Xiangqi Xie, Han Liu, Liang Chen, Zhongsheng Feng, Xiang Gao, Han Gao, Xiaohan Wu, Huiying Lu, Xiaoyu Li, Jinghan Hua, Long Ju, Haifeng Lian, Xue Li, Zhanju Liu

**Affiliations:** ^1^ Center for Inflammatory Bowel Disease Research and Department of Gastroenterology Shanghai Tenth People's Hospital, Tongji University School of Medicine Shanghai China; ^2^ Department of Gastroenterology Qilu Hospital of Shandong University Jinan China; ^3^ Department of Gastroenterology Qilu Hospital (Qingdao), Cheeloo College of Medicine, Shandong University Qingdao China; ^4^ Department of Gastroenterology The Affiliated Yantai Yuhuangding Hospital of Qingdao University Yantai China; ^5^ Department of Big Data in Health Science School of Public Health and The Second Affiliated Hospital, Zhejiang University School of Medicine Hangzhou China

**Keywords:** farnesoid X receptor, inflammatory bowel disease, mucosal homeostasis, neutrophil

## Abstract

Neutrophils significantly accumulate within the inflamed intestinal mucosa of patients with inflammatory bowel disease (IBD), where the farnesoid X receptor (FXR) is typically downregulated. However, the mechanisms by which FXR modulates neutrophil‐mediated mucosal inflammation in IBD remain elusive. Here, we demonstrated that FXR expression is markedly decreased in neutrophils from patients with active IBD. *Fxr* ^−/−^ mice exhibited exacerbated colitis following DSS insults or *Citrobacter rodentium* infection, evidenced by heightened neutrophil‐driven immune responses including increased neutrophil infiltration and neutrophil extracellular trap (NET) formation. Adoptive transfer of *Fxr* ^−/−^ neutrophils into WT recipients exacerbated DSS‐induced intestinal inflammation, indicating that FXR suppresses the pathogenic activity of neutrophils in a neutrophil‐intrinsic manner. An ex vivo functional assay revealed that *Fxr* ^−/−^ neutrophils display elevated ROS production, NET formation, and migratory capacity upon inflammatory challenge. Mechanistically, RNA‐sequencing and functional assays revealed enhanced mTORC1 signaling and glycolysis in *Fxr* ^−/−^ neutrophils. Consistently, pharmacological activation of FXR with INT‐747 significantly restrained the mTORC1‐glycolysis‐mediated proinflammatory responses in neutrophils from IBD patients. Our findings identify FXR as a critical regulator of neutrophil‐mediated mucosal inflammation via the mTORC1‐glycolysis pathway, highlighting its therapeutic potential in IBD.

## Graphical abstract

FXR signaling attenuates neutrophil functions by suppressing mTORC1 activity to ameliorate intestinal mucosal inflammation in IBD. In the inflamed intestinal mucosa, FXR expression is markedly diminished in neutrophils, attenuating its restraint on the mTORC1‐glycolysis axis. This metabolic shift toward elevated glycolysis augments neutrophil migratory capacity, degranulation, and NET formation, contributing to the progression of IBD.

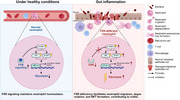

## 1 Introduction

Inflammatory bowel disease (IBD) comprises chronic and relapsing disorders of the gastrointestinal tract, characterized by dysregulated mucosal immunity. Crohn's disease (CD) and ulcerative colitis (UC) are the two major forms of IBD, known for their chronicity, recurrent nature, and often refractory response to treatment. The global incidence of IBD has risen markedly over the past decades and is projected to further increase with population growth and aging [1], underscoring the urgent need to elucidate its pathogenesis and identify new therapeutic targets.

Neutrophils constitute a frontline defense of the innate immune system, rapidly migrating to sites of mucosal injury to eliminate pathogens and provide initial defense against inflammation [2]. Depletion of neutrophils exacerbates colitis induced by dextran sulfate sodium (DSS) and *Citrobacter rodentium* [3]. Our previous data have shown that CD177^+^ neutrophils represent a subset of functionally activated neutrophils characterized by enhanced antimicrobial capacity. These specialized neutrophils demonstrate augmented IL‐22 production, thus facilitating intestinal epithelial repair and exerting a protective role in the pathogenesis of IBD [4]. Recent studies have indicated that neutrophils play both protective and destructive roles in the progression of IBD within distinct inflammatory milieus. In the context of excessive inflammatory signals, neutrophils migrate and accumulate at inflamed sites, where they become hyperactivated and release abundant inflammatory mediators including proinflammatory cytokines, chemokines, myeloperoxidase (MPO), and reactive oxygen species (ROS). This process further recruits additional immune cells (e.g., macrophages, dendritic cells, and T cells), thereby amplifying intestinal mucosal inflammation and delaying mucosal recovery, leading to immunopathological damage [5]. Our previous study has demonstrated that butyrate, a gut microbiota‐derived metabolite, attenuates excessive neutrophil inflammatory activity in the intestinal mucosa of IBD patients, thereby alleviating intestinal inflammation [6]. However, the precise mechanisms by which neutrophil regulates gut inflammation and intestinal homeostasis are still not fully understood. Therefore, comprehensive investigations into neutrophil functions and its roles in the development of intestinal inflammation, coupled with the identification of suitable therapeutic targets, hold significant promise for advancing IBD treatment.

Bile acids are pivotal metabolites produced by host‐gut microbiota interactions and play a crucial role in the pathogenesis of IBD. Primary bile acids (PBAs) synthesized in the liver are converted into secondary bile acids (SBAs) by gut microbiota upon entering the intestine [7, 8]. The farnesoid X receptor (FXR), encoded by *NR1H4*, is a canonical bile acid receptor predominantly expressed in the liver and intestine [9]. Recent studies have demonstrated that bile acid‐FXR signaling is essential for maintaining intestinal epithelial barrier integrity [10, 11, 12]. Moreover, it also modulates the immune responses of dendritic cells and macrophages, thereby mitigating intestinal inflammation [13, 14]. Metabolomic and metagenomic analyses of fecal samples from IBD patients and healthy controls (HC) have revealed higher levels of PBAs, such as cholic acid and chenodeoxycholic acid (CDCA), in IBD patients, whereas the levels of SBAs, such as lithocholic acid and deoxycholic acid, are reduced, which is attributed to the dysbiosis of gut microbiota [15, 16]. Additionally, significant downregulation of FXR expression has been observed in the intestinal tissues of IBD patients [17], and FXR‐null (*Fxr* ^−/−^) mice exhibit increased susceptibility to intestinal inflammation compared with wild‐type (WT) mice [18]. These findings collectively underscore the pivotal role of bile acid‐FXR signaling in modulating immunopathological processes in IBD. However, the precise mechanisms by which FXR signaling influences neutrophil immune responses in IBD remain unclear.

In this study, we investigated the potential roles of FXR in modulating the immune responses of neutrophils during gut inflammation. We observed a significant decrease in FXR expression in neutrophils from IBD patients. *Fxr^−/−^* mice developed exacerbated colitis upon DSS insults or *C. rodentium* infection compared with WT controls, characterized by heightened mucosal infiltration of neutrophils and augmented production of neutrophil extracellular traps (NETs). *Fxr^−/−^* neutrophils displayed increased ROS and MPO release, and heightened NET formation upon inflammatory stimulation via the mechanistic target of rapamycin complex 1 (mTORC1) signaling. Intriguingly, treatment with the FXR agonist INT‐747 significantly attenuated proinflammatory mediator production by lipopolysaccharide (LPS)‐stimulated neutrophils from IBD patients. Therefore, our findings underscore the pivotal role of FXR in modulating neutrophil functions and curbing the proinflammatory responses, thus alleviating intestinal mucosal inflammation in IBD.

## 2 Results

### 2.1 FXR Expression Is Decreased in Patients With Active IBD

**FIGURE 1:**
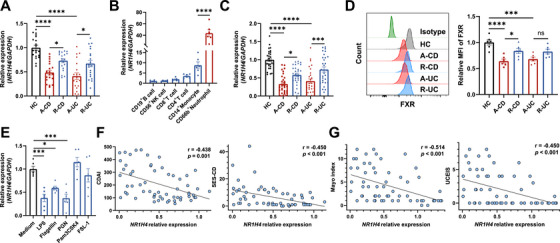
FXR expression is markedly decreased in neutrophils from patients with active IBD. (A) Expression of FXR (encoded by *NR1H4*) in the colonic biopsies from healthy controls (HC, *n* = 20), patients with active CD (A‐CD, *n* = 20), patients with CD in remission (R‐CD, *n* = 20), patients with active UC (A‐UC, *n* = 20), and patients with UC in remission (R‐UC, *n* = 20) was analyzed by qRT‐PCR. (B) CD19^+^ B cells, CD56^+^ NK cells, CD4^+^ T cells, CD8^+^ T cells, CD14^+^ monocytes, and CD66b^+^ neutrophils were isolated from peripheral blood of healthy donors (*n* = 6) using immunomagnetic microbeads and FXR expression was determined by qRT‐PCR. (C) Expression of FXR in peripheral blood neutrophils from HC (*n* = 30), A‐CD (*n* = 30), R‐CD (*n* = 30), A‐UC (*n* = 30), and R‐UC (*n* = 30) was analyzed by qRT‐PCR. (D) FXR expression in peripheral blood neutrophils from six HC, six A‐CD, six R‐CD, six A‐UC, and six R‐UC individuals was analyzed by flow cytometry. Quantitative analysis and representative histogram plot showed mean fluorescence intensity (MFI) of FXR expression. (E) Neutrophils (3 × 10^6^/mL) from healthy donors (*n* = 6) were stimulated with lipopolysaccharide (LPS, 100 ng/mL), Flagellin (100 ng/mL), peptidoglycan (PGN, 1 µg/mL), Pam3CSK4 (1 µg/mL), or fibroblast‐stimulating lipopeptide‐1 (FSL‐1, 100 ng/mL) for 3 h. The expression of FXR was determined by qRT‐PCR. (F–G) Correlations between FXR mRNA levels in peripheral blood neutrophils and disease activity in patients with CD (F, *n* = 60; CDAI: Crohn's disease activity index; SES‐CD: simple endoscopic score for Crohn's disease) or patients with UC (G, *n* = 60; Mayo index; UCEIS, ulcerative colitis endoscopic index of severity). Data were expressed as mean ± SEM. Tukey's test (A–E) and Spearman's correlation analysis (F and G) were used for statistical analysis. *p < 0.05; **p < 0.01; ***p < 0.001; ****p < 0.0001; ns, not significant.

In addition to the alterations in the abundance of bile acids in IBD patients, previous studies have reported reduced FXR signaling but elevated Takeda G protein‐coupled receptor 5 (TGR5, also known as GPBAR1) expression in the colonic tissues from IBD patients [19, 20]. These findings suggest that alterations in bile acid receptors and their functions may contribute to intestinal mucosal inflammation in IBD patients. To further assess the potential roles of bile acid receptors in IBD pathogenesis, we analyzed their expression in human intestinal mucosal biopsies from GEO datasets (GSE75214, GSE165512, and GSE117993). Despite inconsistent outcomes across different datasets, we did observe a significant downregulation of FXR mRNA expression in the intestinal mucosa of patients with active CD and UC (Figure S1A). Furthermore, we harvested intestinal biopsies from HC, active IBD patients and patients in remission, and analyzed the expression levels of different bile acid receptors by qRT‐PCR. The mRNA expression of FXR and vitamin D receptor (VDR) was significantly reduced in the inflamed mucosa of active IBD patients compared with HC, and a notable recovery was seen in those patients at remission stage (Figure 1A, Figure S1B). To explore the contributions of different bile acid receptors to mucosal immunity and their involvement in the pathogenesis of IBD, we assessed the phenotypic expression of various bile acid receptors in distinct immune cell populations. To this end, we isolated CD19^+^ B cells, CD56^+^ NK cells, CD8^+^ T cells, CD4^+^ T cells, CD14^+^ monocytes, and CD66b^+^ neutrophils from peripheral blood of healthy donors using magnetic bead‐based purification. We found that FXR was predominantly expressed in CD66b^+^ neutrophils compared with other immune cell subsets, while TGR5 and VDR showed higher expression in CD14^+^ monocytes (Figure 1B, Figure S1C). A publicly available single‐cell RNA sequencing data [21] from mouse intestinal mucosa further confirmed that neutrophils exhibited the highest FXR expression among all immune cell subsets (Figure S1D). We then validated the expression of FXR in human intestinal neutrophils during IBD through immunofluorescence staining. Consistent with previous studies, almost no neutrophil infiltration was observed in the intestinal mucosa from HC, while the massive recruitment of MPO and FXR double‐positive cells was present in the inflamed intestinal mucosa of IBD patients, further pointing to the expression of FXR in human intestinal neutrophils (Figure S1E). We subsequently conducted correlation analysis between FXR expression levels and different immune cell biomarkers in human intestinal mucosal biopsies from various GEO datasets (GSE75214, GSE165512, and GSE117993) and found a significantly negative correlation between FXR expression and biomarkers associated with neutrophils (CSF3R and S100A8) (Figure S1F). These findings suggest that FXR may maintain intestinal mucosal immune homeostasis by modulating neutrophil functions.

In order to further investigate the alterations in FXR expression in neutrophils of IBD patients, we isolated peripheral blood neutrophils from HC, active IBD patients, and those in remission to assess FXR expression by qRT‐PCR and flow cytometry. Our analysis unveiled a significant decrease in both mRNA and protein levels of FXR in peripheral blood neutrophils from patients with active Crohn's disease (A‐CD) or active ulcerative colitis (A‐UC) compared with those in HC (Figure 1C,D). In contrast, FXR expression was found to be recovered in those patients at the remission stage. Considering that various inflammatory mediators are released into the circulation or targeted organs during inflammatory status, and that neutrophils can be activated by various microbiota‐derived antigens through Toll‐like receptor (TLR) recognition, we further observed that the expression of FXR was decreased upon stimulation with the TLR4 ligand LPS, the TLR5 ligand flagellin, and the TLR2 ligand peptidoglycan (PGN) (Figure 1E). However, the TLR1/2 ligand Pam3CSK4 and the TLR2/6 ligand fibroblast‐stimulating lipopeptide‐1 (FSL‐1) failed to affect FXR expression. Further analysis revealed an inverse correlation between the mRNA levels of FXR in neutrophils and disease activity indices, including the Crohn's Disease Activity Index (CDAI) and the Simple Endoscopic Score for Crohn's Disease (SES‐CD) in patients with CD, and the Mayo index and the Ulcerative Colitis Endoscopic Index of Severity (UCEIS) in patients with UC (Figure 1F,G). Collectively, these data indicate a significant reduction of FXR expression in neutrophils from active IBD patients, which is inversely correlated with disease severity. Therefore, deciphering the detailed mechanistic regulation of FXR on neutrophil functions and its roles in intestinal inflammation is highly warranted.

### 2.2 FXR Deficiency Amplifies Neutrophil‐Driven Intestinal Inflammation

To evaluate the potential role of FXR in colitis development by modulating neutrophil functions, a colitis model was induced in WT mice using DSS in drinking water, and these mice were orally treated with either INT‐747, a selective FXR agonist, or the same volume of solvent as controls (Figure S2A). As expected, WT mice orally administered INT‐747 exhibited milder disease manifestations, as evidenced by reduced weight loss, increased colon length, diminished histological scores, and decreased mRNA levels of proinflammatory mediators in the colonic tissues following exposure to DSS (Figure S2B–E). Flow cytometric analysis revealed a significant reduction in neutrophil infiltration in the colonic tissues of mice treated with INT‐747 compared with vehicle controls (Figure S2F,G). Notably, INT‐747 also effectively suppressed the infiltration of CD4^+^ T cells and F4/80^+^ macrophages in the colonic tissues (Figure S2H,I). Moreover, we also observed a significant reduction in NET formation in the colonic sections from mice treated with INT‐747 compared with controls under DSS induction (Figure S2J). Taken together, these findings indicate that INT‐747 mitigates DSS‐induced colitis, at least partially, by reducing neutrophil‐derived inflammatory mediators.

**FIGURE 2:**
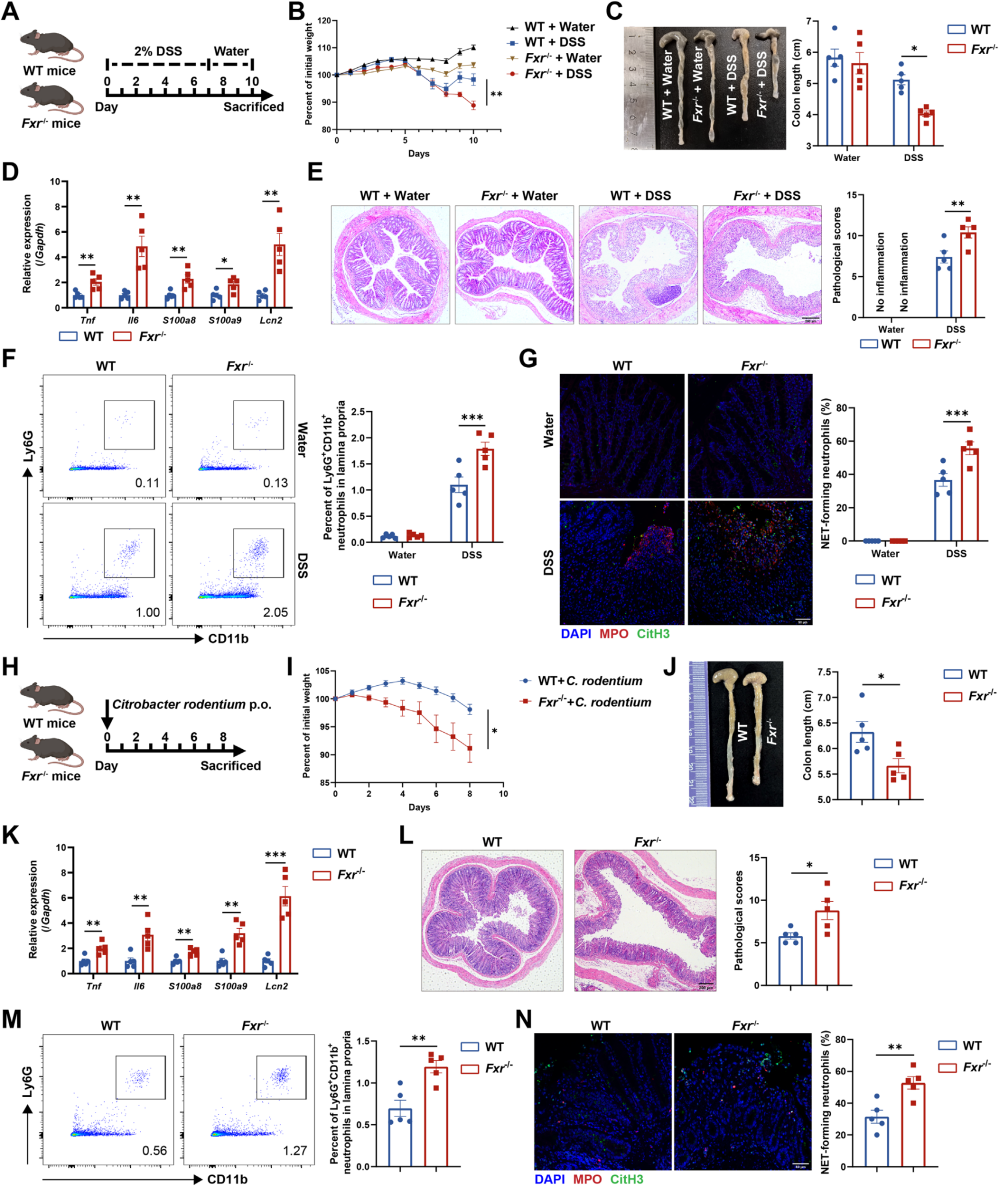
FXR‐deficient mice develop more severe colitis under DSS induction or *Citrobacter rodentium* infection. (A–G) Eight‐week‐old WT and *Fxr* ^−/−^ mice were administered 2% DSS in drinking water for 7 days, followed by another 3 days of regular water consumption to induce colitis (*n* = 5/group). All mice were sacrificed on Day 10. (B) Changes in body weight during the disease course. (C) Representative image and quantification of colon length on Day 10. (D) Expression of inflammatory mediators in the colonic tissues was measured by qRT‐PCR. (E) Hematoxylin and eosin (H&E) staining and histopathological scores were applied to assess the severity of colitis. Scale bars, 200 µm. (F) Flow cytometric analysis of lamina propria‐infiltrating neutrophils. (G) Representative immunofluorescence staining of specific markers for neutrophil extracellular traps (NETs) in the colonic sections, with quantification of NET‐forming neutrophils. Scale bars, 50 µm. (H–N) Eight‐week‐old WT and *Fxr* ^−/−^ mice were orally administered with *Citrobacter rodentium* (*C. rodentium*, 2 × 10^9^ CFU/mouse) after fasting for 10 h on Day 0 to induce colitis (*n* = 5/group). All mice were sacrificed on Day 8. (I) Changes in body weight over the 8‐day modeling period. (J) The gross morphology and length of colon on Day 8. (K) Expression of inflammatory mediators in the colonic tissues was analyzed by qRT‐PCR. (L) H&E staining and histopathological scores were applied to assess the severity of colitis. Scale bars, 200 µm. (M) Flow cytometric analysis of lamina propria‐infiltrating neutrophils. (N) Immunofluorescence staining of NET markers in the colonic sections, with quantification of NET‐forming neutrophils. Scale bars, 50 µm. Data were expressed as mean ± SEM. Tukey's test (B, C, E, F, and G) and Student's *t*‐test (D and I–N) were used for statistical analysis. *p < 0.05; **p < 0.01; ***p < 0.001; ns, not significant.

To understand the critical role of FXR signaling in regulating neutrophil functions in the pathophysiology of intestinal mucosal inflammation, we established a DSS‐induced colitis model in *Fxr^−/−^* mice (Figure 2A). We found that *Fxr^−/−^* mice exhibited exacerbated mucosal inflammation, as evidenced by increased weight loss, shortened colon length, upregulated mRNA expression levels of *Tnf*, *Il6*, *S100a8*, *S100a9*, and *Lcn2* in the colonic tissues, and heightened pathological scores compared with WT controls (Figure 2B–E, Figure S3A). Interestingly, FXR deficiency resulted in a significant increase in neutrophil infiltration within the lamina propria (Figure 2F, Figure S3B). A substantial elevation in NET formation was also observed in the colonic sections of *Fxr^−/−^* mice compared with WT controls under DSS induction (Figure 2G). Moreover, the infiltration of CD4^+^ T cells and F4/80^+^ macrophages was markedly increased in the lamina propria of *Fxr* ^−/−^ mice (Figure S3C,D).

To further verify these findings, we employed a *C. rodentium*‐induced acute colitis model in *Fxr^−/−^* mice (Figure 2H). As expected, *Fxr^−/−^* mice displayed more pronounced weight loss, shortened colon length, higher pathological scores, and increased proinflammatory cytokines (Figure 2I–L, Figure S3E), along with increased infiltrations of neutrophils (Figure 2M, Figure S3F), CD4^+^ T cells, and F4/80^+^ macrophages (Figure S3G,H) in the colonic tissues compared with WT controls. In addition, *Fxr^−/−^* mice exhibited elevated NET formation in the colonic tissues (Figure 2N). Overall, these data suggest that the absence of FXR could exacerbate intestinal inflammation partially via compromising neutrophil immune response.

### 2.3 Neutrophil‐Intrinsic FXR Signaling Protects the Intestinal Mucosa From Inflammatory Injury

**FIGURE 3:**
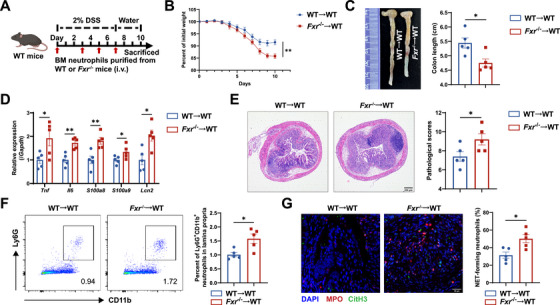
Adoptive transfer of FXR‐deficient neutrophils exacerbates DSS‐induced colitis in mice. (A) Purified bone marrow (BM)‐derived neutrophils isolated from WT or *Fxr* ^−/−^ donors were intravenously transferred (2 × 10^6^/mouse) on Days 0, 3, 5, and 7 (*n* = 5/group). All recipients were administered 2% DSS in drinking water for 7 days, followed by another 3 days of regular water consumption to induce colitis. All mice were sacrificed on Day 10. (B) Changes in body weight over a 10‐day modeling period. (C) The gross morphology and length of colon on Day 10. (D) Expression of inflammatory mediators in the colonic tissues was analyzed by qRT‐PCR. (E) H&E staining and histopathological scores were applied to assess the severity of colitis. Scale bars, 200 µm. (F) Flow cytometric analysis of lamina propria‐infiltrating neutrophils. (G) Representative immunofluorescence staining of NET markers in the colonic sections, with quantification of NET‐forming neutrophils. Scale bars, 30 µm. Data were expressed as mean ± SEM. Student's *t*‐test was used for statistical analysis. *p < 0.05; **p < 0.01; ns, not significant.

To better determine whether the loss of neutrophil‐intrinsic FXR contributes to aggravated intestinal mucosal inflammation, we established an adoptive transfer model [22, 23, 24]. Bone marrow‐derived neutrophils isolated from WT and *Fxr* ^−/−^ mice were intravenously transferred into WT recipients during the course of DSS‐induced colitis on Days 0, 3, 5, and 7 (Figure 3A). Compared with mice receiving WT neutrophils, those receiving *Fxr* ^−/−^ neutrophils developed more severe intestinal inflammation, as evidenced by greater body weight loss, shorter colon length, higher mRNA levels of proinflammatory mediators in the colonic tissues, and higher histopathological scores (Figure 3B–E, Figure S3I). Furthermore, the colons of mice transferred with *Fxr* ^−/−^ neutrophils exhibited increased infiltration of neutrophils (Figure 3F, Figure S3J), CD4^+^ T cells, and F4/80^+^ macrophages (Figure S3K, L). As expected, increased formation of NETs was also observed in the colon of mice receiving *Fxr* ^−/−^ neutrophils compared with controls (Figure 3G). Collectively, these findings demonstrate that neutrophil‐intrinsic FXR signaling plays a crucial protective role in maintaining intestinal immune homeostasis. Loss of FXR in neutrophils enhances their proinflammatory activity, thereby exacerbating mucosal inflammatory injury.

### 2.4 FXR Deficiency Facilitates the Inflammatory Responses in Neutrophils

**FIGURE 4:**
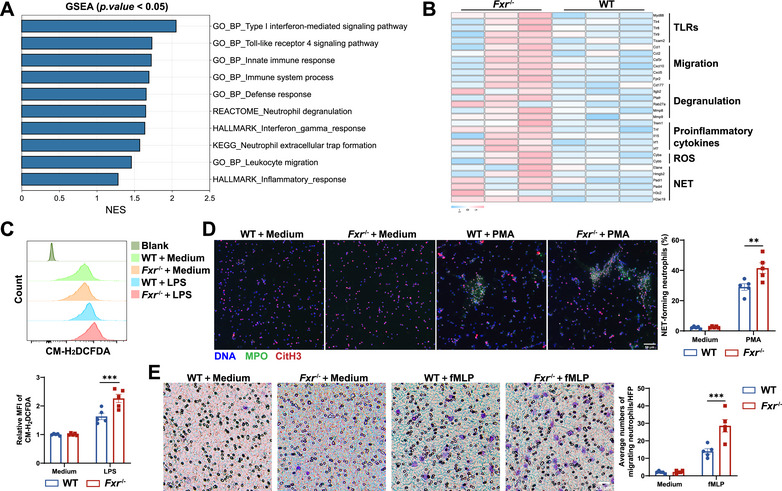
FXR‐deficient neutrophils exhibit augmented proinflammatory activity. BM‐derived neutrophils (*n* = 3/group) were harvested from WT and *Fxr* ^−/−^ mice for RNA sequencing analysis. (A) Gene set enrichment analysis (GSEA) of transcriptomic profiles. NES, normalized enrichment score. (B) Heatmap of genes associated with neutrophil functions. TLRs, Toll‐like receptors; ROS, reactive oxygen species. (C) Representative histogram and quantification of MFI of the ROS probe CM‐H_2_DCFDA in BM‐derived neutrophils (1 × 10^6^/mL) after 3 h stimulation with LPS (300 ng/mL) (*n* = 5/group). (D) Representative co‐staining of DNA, citrullinated histone‐3 (CitH3), and myeloperoxidase (MPO) to assess NET formation in BM‐derived neutrophils (1 × 10^6^/mL) after treatment with phorbol 12‐myristate 13‐acetate (PMA, 100 ng/mL) for 3 h (*n* = 5/group). Scale bars, 50 µm. Quantification of NET‐forming neutrophils is shown in the bar chart. (E) Neutrophil migration was assessed using a Transwell assay, in which BM‐derived neutrophils (1 × 10^6^/mL) were seeded in the upper chamber and stimulated with N‐formyl‐methionyl‐leucyl‐phenylalanine (fMLP, 50 nM) in the lower chamber for 2 h (*n* = 5/group). Migrating neutrophils were quantified per high‐power field (HPF). Scale bars, 25 µm. Data were expressed as mean ± SEM. Tukey's test was used for statistical analysis. **p < 0.01; ***p < 0.001; ns, not significant.

To better understand how FXR regulates neutrophil functions in vitro, we isolated bone marrow‐derived neutrophils from both WT and *Fxr^−/−^* mice and conducted RNA sequencing analysis. A total of 566 differentially expressed genes were identified between these two groups (Figure S4A). Pathway analysis revealed significant enrichment of pathways related to innate immune response, neutrophil degranulation, and NET formation in *Fxr^−/−^* neutrophils (Figure 4A, Figure S4B,C). Notably, genes associated with neutrophil degranulation, ROS release, neutrophil migration, NET formation, and TLRs were upregulated in *Fxr^−/−^* neutrophils (Figure 4B). We further verified the elevated levels of *Tnf*, *Il6*, *S100a8*, and *Lcn2* in *Fxr^−/−^* neutrophils under LPS‐stimulated conditions by qRT‐PCR (Figure S4D). ROS and MPO production were also significantly higher in LPS‐treated *Fxr^−/−^* neutrophils compared with WT controls (Figure 4C, Figure S4E,F). Given that MPO is a bactericidal component of NETs, we further investigated the formation of NETs in neutrophils under FXR‐deficient conditions. As expected, we found that the formation of NETs was significantly increased in *Fxr^−/−^* neutrophils compared with WT controls (Figure 4D). Moreover, we found that *Fxr^−/−^* neutrophils showed an enhanced migratory capacity compared with WT controls (Figure 4E) through a Transwell assay. These findings suggest that FXR plays an important role in orchestrating the immunoregulation of neutrophils and that its deficiency facilitates the proinflammatory responses of neutrophils.

### 2.5 mTORC1‐Glycolysis Pathway Mediates the Immunomodulatory Effects of FXR in Neutrophils

**FIGURE 5:**
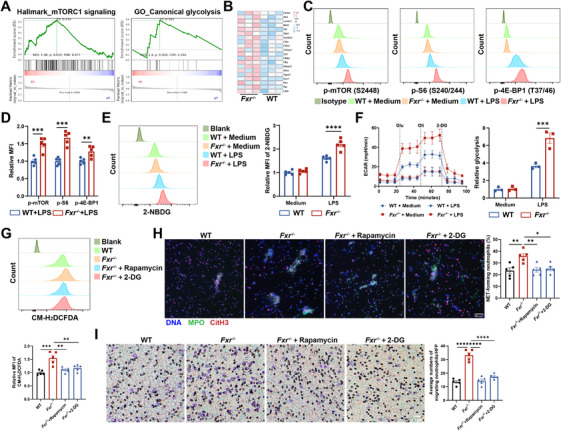
FXR suppresses proinflammatory functions in neutrophils through the mTORC1 pathway. (A) GSEA analysis of the transcriptomic data shown in Figure 4. (B) Heatmap of genes involved in the mTORC1‐glycolysis pathway. (C and D) Phospho‐flow analysis of mTORC1 substrates in BM‐derived neutrophils (3 × 10^6^/mL) under 3 h of LPS (300 ng/mL) treatment (*n* = 5/group). P‐mTOR, phospho‐mTOR; p‐S6, phospho‐S6; p‐4E‐BP1, phospho‐4E‐BP1. (E) Representative histogram and quantification of 2‐NBDG fluorescence intensity, a fluorescent glucose analog used to monitor glucose uptake, in BM‐derived neutrophils (3 × 10^6^/mL) after 3 h of LPS (300 ng/mL) stimulation (*n* = 5/group). (F) BM‐derived neutrophils (2 × 10^6^/mL) were treated with or without LPS (300 ng/mL) for 3 h, and the extracellular acidification rate (ECAR) was measured using a Seahorse analyzer (*n* = 3/group). Glu, glucose; Oli, oligomycin; 2‐DG, 2‐Deoxy‐D‐glucose. (G) BM‐derived neutrophils (1 × 10^6^/mL) were treated with LPS (300 ng/mL) in the presence or absence of rapamycin (2 µM) or 2‐DG (5 mM) for 3 h. ROS production was determined by flow cytometry using CM‐H_2_DCFDA (*n* = 5/group). (H) BM‐derived neutrophils (1 × 10^6^/mL) were treated with PMA (100 ng/mL) in the presence or absence of rapamycin (2 µM) or 2‐DG (5 mM) for 3 h. NET formation was detected by immunofluorescence staining. Scale bars, 50 µm. Quantification of NET‐forming neutrophils is shown in the bar chart (*n* = 5/group). (I) BM‐derived neutrophils (1 × 10^6^/mL) pretreated with or without rapamycin (2 µM) or 2‐DG (5 mM) were placed in the upper chamber of the Transwell insert, and fMLP (50 nM) was added to the lower chamber for 2 h (*n* = 5/group). The migration of neutrophils was detected as described in Figure 4E. Scale bars, 25 µm. Data were expressed as mean ± SEM. Tukey's test (E–I) and Student's *t*‐test (D) were used for statistical analysis. *p < 0.05; **p < 0.01; ***p < 0.001; ****p < 0.0001; ns, not significant.

To elucidate the underlying mechanisms by which FXR modulates the immune responses of neutrophils, we conducted further analysis of the transcriptome data. Gene set enrichment analysis (GSEA) showed significant enrichment of mTORC1 signaling, HIF‐1 signaling, and canonical glycolysis pathways in *Fxr^−/−^* neutrophils (Figure 5A, Figure S5A). Additionally, genes associated with mTORC1 and glycolysis were significantly upregulated in *Fxr^−/−^* neutrophils (Figure 5B). To bolster these findings, we isolated bone marrow‐derived neutrophils from *Fxr^−/−^* and WT mice and treated them with LPS in vitro. We then quantified mTOR pathway activity using intracellular phospho‐flow, measuring phosphorylation of mTOR (Ser2448), eIF4E‐binding protein 1 (4E‐BP1; Thr37/46), and ribosomal protein S6 (rpS6, hereafter “S6”; Ser240/244) as readouts of mTORC1 activity, and phosphorylation of protein kinase B (Akt; Ser473) as readout of mTORC2 activity. Consistently, *Fxr^−/−^* neutrophils exhibited increased phosphorylation of mTOR, S6, and 4E‐BP1 compared with WT neutrophils upon LPS treatment in vitro (Figure 5C,D). Conversely, Akt phosphorylation in neutrophils was minimally affected by FXR deficiency (Figure S5B). We then observed that the mRNA levels of *Hif1a*, *Slc2a4*, *Hk3*, and *Pdk4* were elevated in *Fxr^−/−^* neutrophils compared with controls under LPS‐stimulated conditions (Figure S5C). Moreover, we found that *Fxr^−/−^* neutrophils showed significantly increased glucose uptake, as assessed by flow cytometry using the fluorescent glucose analog 2‐NBDG (Figure 5E). To further verify the changes in glycolysis, we measured the extracellular acidification rate (ECAR) using an extracellular flux Seahorse analyzer. Consistently, *Fxr^−/−^* neutrophils exhibited enhanced ECAR, an indicator of increased glycolysis, compared with WT controls (Figure 5F, Figure S5D). Subsequently, we investigated whether the mTORC1 signaling and glycolysis are involved in FXR regulation of neutrophil functions. We treated *Fxr^−/−^* and WT neutrophils with rapamycin, an inhibitor of the mTORC1 signaling, and 2‐deoxy‐D‐glucose (2‐DG), an inhibitor of glycolysis, respectively, and found that inclusion of either rapamycin or 2‐DG markedly reversed the excessive release of ROS, MPO, NETs, and exorbitant migratory capacity of *Fxr^−/−^* neutrophils (Figure 5G–I, Figure S5E,F). Furthermore, rapamycin treatment could rescue the elevated glycolysis observed in *Fxr^−/−^* neutrophils induced by LPS compared with WT controls (Figure S5G–I). Therefore, these results indicate that FXR modulates immune responses of neutrophils through the mTORC1‐glycolysis pathway.

To further substantiate that FXR suppresses proinflammatory phenotypes of neutrophils via the mTORC1‐glycolysis pathway, we tested this mechanism in vivo using a colitis model. Specifically, we employed WT and *Fxr^−/−^* mice subjected to DSS‐induced colitis and pharmacologically modulated the mTORC1‐glycolysis axis by daily administration of either rapamycin or 2‐DG (Figure S6A). Remarkably, *Fxr^−/−^* mice treated with rapamycin or 2‐DG displayed less body weight loss, increased colon length, lowered expression of proinflammatory mediators, improved histopathological scores, reduced neutrophil infiltration, and diminished NET formation in the colonic tissues, compared with untreated *Fxr^−/−^* mice (Figure S6B–G). Collectively, these findings provide in vivo evidence that FXR alleviates intestinal mucosal inflammation by constraining neutrophil hyperactivation through the mTORC1‐glycolysis signaling pathway.

### 2.6 FXR Signaling Inhibits the Proinflammatory Phenotypes of Neutrophils From IBD Patients

**FIGURE 6:**
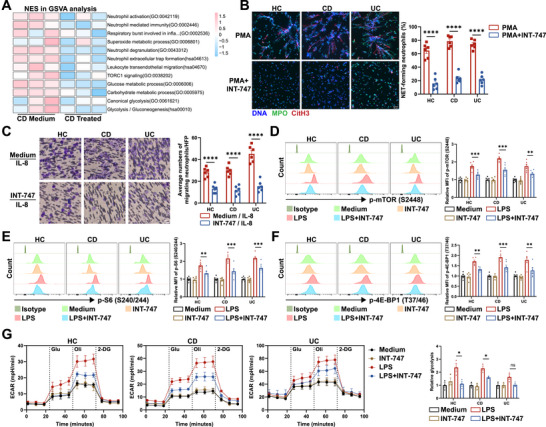
FXR signaling suppresses proinflammatory functions in neutrophils from IBD patients. (A) Peripheral blood (PB) neutrophils (3 × 10^6^/mL) were isolated from patients with active CD (*n* = 3) and treated with or without INT‐747 (40 µM) for 3 h. Total RNA was extracted and subjected to RNA sequencing, followed by gene set variation analysis (GSVA) to assess transcriptomic changes. (B) Representative co‐staining of DNA, CitH3, and MPO to assess NET formation in PB neutrophils (1 × 10^6^/mL) isolated from healthy donors (HC, *n* = 6), patients with active CD (*n* = 6) and patients with active UC (*n* = 6) after 3 h of PMA (100 ng/mL) stimulation in the presence or absence of INT‐747 (40 µM). Scale bars, 50 µm. Quantification of NET‐forming neutrophils is shown in the bar chart. (C) PB neutrophils (1 × 10^6^/mL) isolated from healthy donors (HC, *n* = 6), patients with active CD (*n* = 6), and patients with active UC (*n* = 6) were pretreated with or without INT‐747 (40 µM) and seeded in the upper chamber of the Transwell insert. IL‐8 (20 ng/mL) was added to the lower chamber to induce migration for 2 h. The migrated neutrophils were quantified as described in Figure 4E. Scale bars, 25 µm. (D–F) PB neutrophils (3 × 10^6^/mL) isolated from healthy donors, patients with active CD, and patients with active UC were stimulated with LPS (300 ng/mL) in the presence or absence of INT‐747 (40 µM) for 3 h. Phosphorylation of mTORC1 substrates was analyzed by phospho‐flow cytometry (*n* = 6/group). (G) ECAR levels in neutrophils treated as in (D–F) were measured using a Seahorse analyzer (*n* = 3/group). Glu, glucose; Oli, oligomycin. Data were expressed as mean ± SEM. Student's *t*‐test (B and C), and Tukey's test (D–G) were used for statistical analysis. *p < 0.05; **p < 0.01; ***p < 0.001; ****p < 0.0001; ns, not significant.

Since insufficient FXR in neutrophils rendered mice more susceptible to experimental colitis and exacerbated inflammatory responses in *Fxr^−/−^* neutrophils, we next explored the impact of FXR signaling on neutrophils from IBD patients. INT‐747, a synthetic analog derived from CDCA, demonstrates two to five times higher potency in activating FXR signaling [25]. We utilized it to investigate the regulatory effects of bile acid‐FXR signaling on neutrophil functions. Neutrophils were isolated from both active IBD patients and HC, treated with or without INT‐747, and then subjected to RNA sequencing. Interestingly, INT‐747‐treated neutrophils exhibited significant differences in gene expression profiles compared with untreated cells (Figure S7A). As expected, freshly isolated neutrophils from active CD or UC patients showed increased inflammatory responses compared with those from HC (Figure S7B). Pathway analysis further revealed that INT‐747‐induced functional changes were characterized by reduced neutrophil activation, degranulation, and NET formation (Figure 6A; Figure S7C,D). Accordingly, genes associated with neutrophil degranulation, migration, and superoxide generation showed reduced expression with INT‐747 treatment (Figure S7E).

Subsequently, we isolated neutrophils from HC and patients with active IBD and treated them with INT‐747 to assess the effect of FXR signaling on their inflammatory responses. As expected, INT‐747 treatment markedly downregulated the expression of proinflammatory cytokines, including TNF‐α, IL‐1β, and IL‐6 (Figure S8A,B). In addition, the expression of S100A8, S100A9, and LCN2, key biomarkers associated with IBD disease severity, was significantly reduced following INT‐747 treatment (Figure S8C,D). INT‐747 also suppressed the expression of the chemokine IL‐8 (Figure S8E,F). Moreover, INT‐747 profoundly inhibited the production of ROS and MPO (Figure S8G,H), and markedly diminished phorbol 12‐myristate 13‐acetate (PMA)‐induced NET formation (Figure 6B). Furthermore, the Transwell assay revealed that INT‐747 significantly attenuated the migratory capacity of neutrophils from IBD patients (Figure 6C).

As described in the RNA sequencing data among *Fxr^−/−^* and WT neutrophils, gene set variation analysis (GSVA) revealed that TORC1 signaling and canonical glycolysis pathways were downregulated in IBD neutrophils (particularly in CD patients) treated with INT‐747 (Figure 6A, Figure S7D). Flow cytometry analysis further revealed that INT‐747 significantly inhibited LPS‐induced phosphorylation of mTORC1 pathway components, including mTOR (Ser2448), 4E‐BP1 (Thr37/46), and S6 (Ser240/244) (Figure 6D–F). Additionally, INT‐747 markedly suppressed glycolysis in IBD neutrophils (Figure 6G, Figure S9A,B). Taken together, these findings unequivocally substantiate a critical role of FXR signaling in regulating mucosal neutrophil functions during IBD, further corroborating the key findings from the murine colitis model treated by INT‐747.

## 3 Discussion

Previous studies have shown that IBD patients are featured with reduced bile acid‐dependent FXR activation and that the suppression of FXR signaling could disrupt intestinal epithelial barrier integrity [20, 26, 27]. Importantly, increasing lines of evidence have demonstrated the regulatory role of FXR in immune responses of various immune cells (e.g., macrophages and T lymphocytes) [28, 29], while its specific role in regulating neutrophil functions in the pathogenesis of IBD remains unclear. In this study, we found diminished FXR expression in IBD neutrophils and most interestingly observed that the FXR signaling participates in the regulation of murine colitis by modulating neutrophil‐mediated inflammatory responses in the intestinal mucosa. Notably, the use of FXR agonist INT‐747 effectively suppressed the release of proinflammatory mediators from neutrophils. Mechanistically, FXR mitigated neutrophil‐driven inflammation via the mTORC1‐glycolysis pathway.

Bile acids are pivotal products of host‐gut microbiota interactions, exerting diverse biological effects. FXR, which exhibits strong affinity for several major endogenous bile acids, predominantly localizes within intestinal epithelial cells and governs bile acid enterohepatic circulation. Additionally, it regulates the expression of tight junction proteins and the renewal of intestinal epithelial cells [10, 30, 31, 32]. While its presence in immune cells is typically subdued, FXR may modulate diverse functions among different immune cells [18, 33, 34]. In this study, we identified heightened FXR expression in neutrophils compared with other immune cell subsets. Moreover, the expression levels of FXR in neutrophils were inversely correlated with disease severity of IBD patients. We established murine colitis models to further investigate the potential role of FXR in modulating intestinal inflammation through neutrophils, and found that oral administration of the FXR agonist INT‐747 significantly restricted the infiltration of immune cells in the intestinal mucosa and attenuated intestinal inflammation, aligning with previous reports [14]. Interestingly, our study identified that INT‐747 treatment led to a marked decrease in neutrophil infiltration and inhibited the release of NETs in intestinal tissues. In contrast, *Fxr^−/−^* mice developed a more severe inflammatory phenotype, with increased colonic infiltration of CD4^+^ T cells, F4/80^+^ macrophages, and neutrophils, along with augmented mucosal NET formation, compared with WT controls. Importantly, adoptive transfer experiments further confirmed the essential role of neutrophil‐intrinsic FXR in mucosal protection. When *Fxr^−/−^* neutrophils were transferred into WT recipients undergoing DSS‐induced colitis, these mice developed more severe intestinal inflammation than those receiving WT neutrophils. Taken together, these results provide compelling evidence that loss of FXR within neutrophils enhances their proinflammatory activity, thereby aggravating mucosal inflammation and accelerating colitis progression.

Accumulating evidence indicates that FXR modulates diverse immune cell functions, including suppressing proinflammatory cytokine and chemokine expression in human CD14^+^ monocytes and dendritic cells [14], promoting M2 polarization in bone marrow‐derived macrophages [35] and restraining intestinal innate lymphoid cells expansion and production of IL‐17 [36]. To investigate the regulatory role of FXR in neutrophil functions, we conducted RNA sequencing analysis on bone marrow‐derived neutrophils from *Fxr^−/−^* and WT mice, confirming the immunomodulatory effects of FXR on neutrophils. Subsequent in vitro experiments further revealed that FXR‐deficient neutrophils exhibited increased release of ROS, MPO, and NETs upon inflammatory stimulation, along with enhanced migratory capacity, compared with WT controls.

Glycolysis provides essential energy and metabolic intermediates for neutrophil activation and effector functions, and mTORC1 serves as a key regulator by promoting HIF‐1α‐mediated transcription of glycolytic enzymes and controls glucose uptake and utilization [37, 38, 39]. In our RNA sequencing data, we did observe an upregulation of glycolysis and the mTORC1 pathway in *Fxr^−/−^* neutrophils. Treatment with 2‐DG, a well‐established glycolysis inhibitor, significantly reversed the hyperactive phenotypes in *Fxr^−/−^* neutrophils, consistent with previous studies demonstrating the involvement of glycolysis in ROS and MPO release, NET formation, and migration [40, 41, 42]. Furthermore, the upregulation of glycolysis levels in activated *Fxr^−/−^* neutrophils showed significant restoration following the inhibition of the mTORC1 pathway by rapamycin. These results are consistent with previous findings showing that FXR activation inhibits the mTORC1 pathway in liver progenitor cells of regenerating livers and decreases glucagon‐like peptide‐1 secretion by inhibiting glycolysis in enteroendocrine L cells [43, 44]. Importantly, our in vivo findings further substantiate this regulatory axis within the context of intestinal inflammation. Pharmacological inhibition of either mTORC1 signaling or glycolysis markedly ameliorated disease severity in *Fxr^−/−^* mice subjected to DSS‐induced colitis by restraining neutrophil infiltration and NET formation. Taken together, these findings uncover a distinct mechanism whereby FXR modulates neutrophil functions by suppressing the mTORC1‐glycolysis pathway. By curbing metabolic overactivation, FXR prevents the transition of neutrophils from host‐protective to tissue‐damaging states during intestinal inflammation.

As a major intracellular bile acid receptor and a key regulator of bile acid homeostasis, as well as lipid and glucose metabolism, FXR has emerged as a pivotal therapeutic target for cholestatic and fatty liver diseases [45]. Ocaliva, a steroidal FXR agonist and the first‐in‐class therapeutic FXR ligand, has received conditional approval as the second‐line therapy for primary biliary cholangitis by both the US Food and Drug Administration and the European Medicine Agency [46, 47]. Other published FXR agonists, including tropifexor [48], cilofexor [49], and vonafexor [50], have undergone human clinical trials. Despite the promising anti‐inflammatory effects observed in murine models, the clinical utility of FXR agonists in treating IBD remains limited. To elucidate the potential therapeutic value of the FXR signaling in IBD, we treated IBD neutrophils with the FXR agonist INT‐747 and observed that activation of FXR signaling could markedly downregulate the immune response of neutrophils, indicating a potential protective role in restraining intestinal mucosal inflammation. While our study highlights the immunomodulatory potential of FXR in neutrophils, it is limited by reliance on ex vivo neutrophils from IBD patients and murine colitis models. Future studies focusing on pharmacological strategies that selectively target neutrophil‐derived FXR within the intestinal microenvironment in clinical settings will be essential to translate FXR‐based interventions into therapeutic benefits for IBD patients.

In summary, our study demonstrates that the FXR signaling exerts a direct immunomodulatory effect on neutrophils and plays a protective role in intestinal mucosal inflammation. We identified a critical role of FXR in modulating neutrophil functions by reducing the production of proinflammatory mediators and inhibiting the migratory capacity via the mTORC1‐glycolysis pathway, thus aiding in the mitigation of intestinal inflammation. Consequently, pharmacologic activation of FXR may provide a novel therapeutic avenue by modulating neutrophil functions for IBD.

## 4 Materials and Methods

### 4.1 Reagents

The details of reagents, including chemical compounds and antibodies, used in this study are listed in Table S1.

### 4.2 Subjects

All healthy volunteers and IBD patients were enrolled from the Department of Gastroenterology, Shanghai Tenth People's Hospital of Tongji University (Shanghai, China) from July 2020 to June 2023. The diagnoses of CD and UC were established through a combination of clinical manifestations, endoscopic findings, radiological assessments, and histological features with exclusions for autoimmune diseases, infections, and tumors [51, 52, 53]. Endoscopic biopsies were obtained from the aforementioned participants undergoing diagnostic or therapeutic endoscopy. Detailed clinical characteristics of patients with IBD and HC are provided in Table S2.

### 4.3 Mice

WT C57BL/6 mice were purchased from Shanghai SLAC Laboratory Animal Co., Ltd (Shanghai, China). *Fxr* ^−/−^ mice were kindly provided by Li Yang (Shanghai Key Laboratory of Complex Prescriptions and MOE Key Laboratory for Standardization of Chinese Medicines, Shanghai University of Traditional Chinese Medicine) [54]. Primers used to test the presence of *Fxr* were: 5′‐ACAGCGTGGAAAGCAACAG‐3′ and 5′‐GTCCTCACGGAAAGCTCTTACA‐3′. All mice utilized in this investigation were male, aged 8–10 weeks, and weighing 20–25 g. They were raised and housed in specific pathogen‐free conditions at the Experimental Animal Center of Tongji University School of Medicine (Shanghai, China).

### 4.4 Isolation of Neutrophils

For human peripheral blood neutrophil isolation, peripheral blood samples were subjected to density gradient centrifugation using Ficoll‐Paque PLUS density gradient media and neutrophils were obtained from the bottom layer after hypotonic lysis of red blood cells with BD Pharm Lyse Lysing Buffer. The isolation of neutrophils from the bone marrow of mice was performed using a murine neutrophil isolation kit according to the manufacturer's instructions (Miltenyi Biotec; Bergisch Gladbach, Germany).

### 4.5 DSS‐Induced Colitis in Mice

WT and *Fxr* ^−/−^ mice were subjected to a 7‐day oral administration of 2% DSS in drinking water, followed by a switch to normal drinking water for an additional 3 days, as described previously [55]. The mice were euthanized for analysis on Day 10. For INT‐747 treatment, WT mice were orally administered INT‐747 (5 mg/kg) daily for 10 days, with a switch to drinking water containing 2% DSS from the third day onwards. Mice in the control group received an equivalent volume of vehicle by oral gavage. For adoptive transfer experiments, purified bone marrow neutrophils derived from WT or *Fxr* ^−/−^ donor mice were intravenously transferred (2 × 10^6^/mouse) into WT recipients on Days 0, 3, 5, and 7. For manipulating the mTORC1 signaling, *Fxr* ^−/−^ mice received daily oral gavage of rapamycin (1.5 mg/kg). To inhibit glycolysis, *Fxr* ^−/−^ mice received daily intraperitoneal injections of 2‐DG (500 mg/kg).

### 4.6 *C. rodentium* Infection Model in Mice

*C. rodentium* strain DBS100 (ATCC51459) was purchased from American Type Culture Collection (ATCC; Rockefeller, MD, USA). The *C. rodentium*‐induced murine colitis model was established as described previously [3]. In brief, *C. rodentium* was cultured by shaking overnight in Luria‐Bertani broth at 37°C. The bacterial suspension was then serially diluted and plated onto MacConkey agar plates to determine the optimal colony‐forming unit (CFU). WT and *Fxr* ^−/−^ mice received oral administration of *C. rodentium* (2 × 10^9^ CFU/mouse) after fasting for 10 h on Day 0. Mice were euthanized on Day 8 post‐infection.

### 4.7 Flow Cytometry

For staining neutrophils, CD4^+^ T cells, and macrophages within the lamina propria, lamina propria mononuclear cells were obtained as previously described [56] and incubated with fluorochrome‐conjugated antibodies against cell‐surface markers for 30 min at 4°C. Live/Dead Fixable Dead Cell Stain Kit (Invitrogen; Eugene, OR, USA) was used to exclude dead cells. For intracellular staining of FXR, peripheral blood neutrophils were permeabilized with the Transcription Factor Staining Buffer Set, then incubated with the anti‐FXR antibody for 1 h at room temperature and subsequently labeled with a fluorescent secondary antibody. For intracellular staining of phospho‐proteins, cells were fixed with IC Fixation Buffer and then permeabilized with 90% methanol followed by staining with phospho‐specific antibodies for 1 h at room temperature. For intracellular ROS detection, cells were incubated for 30 min at 37°C with 10 µM CM‐H_2_DCFDA. For glucose uptake detection, cells were incubated for 30 min at 37°C with the glucose analog 2‐NBDG (100 µM). Following these, BD FACSCanto II Flow Cytometer (BD Biosciences; San Diego, CA, USA) was utilized to analyze the samples, and all data were analyzed using FlowJo software (version 10.8.1; BD Biosciences; Ashland, OR, USA).

### 4.8 Immunofluorescence Staining of NETs in the Colonic Tissues

The colonic tissues were fixed in 4% paraformaldehyde and embedded in paraffin. Sections (5 µm) were prepared and mounted on glass slides. Then the slides were stained using the TSAPlus fluorescence staining kit (Servicebio; Wuhan, China) with anti‐CitH3 and anti‐MPO antibodies according to the manufacturer's instructions. DAPI was used to counterstain the DNA. Images were acquired using Pannoramic SCAN II (3DHISTECH; Budapest, Hungary). The percentage of NET‐forming neutrophils was defined as the ratio of CitH3 and MPO double‐positive neutrophils to the total MPO‐positive neutrophils [57].

### 4.9 RNA‐Sequencing Analysis

Total RNA extracted from neutrophils was used for library construction with the VAHTS Universal V6 RNA‐seq Library Prep Kit (Vazyme; Nanjing, China) according to the manufacturer's instructions, and sequencing was performed on the Illumina Novaseq 6000 platform (Illumina; San Diego, CA, USA). Raw reads were then processed with fastp, mapped to the reference genome using HISAT2, and the read counts of each gene were generated by HTSeq‐count. Differentially expressed genes were identified by DESeq2, with *p* value < 0.05 and fold change > 1.5 or < 0.67. For Gene Ontology (GO) and Kyoto Encyclopedia of Genes and Genomes (KEGG) enrichment analysis, differentially expressed genes were implemented by the clusterProfiler package. GSEA was carried out using the GSEA software, while GSVA was conducted with the GSVA package in R. Data visualization, including volcano plots, bar charts, bubble plots, and heatmaps, was performed in R (v 3.2.0).

### 4.10 Analysis of NET Formation of Neutrophils In Vitro

Neutrophils (1 × 10^6^/mL) were seeded on the poly‐L‐lysine‐coated glass slides and stimulated with PMA (100 ng/mL) for 3 h at 37°C. They were then fixed with 4% paraformaldehyde, permeabilized with 0.3% Triton X‐100, and blocked with 10% normal goat serum. After overnight incubation with anti‐CitH3 and anti‐MPO antibodies at 4°C, the slides were washed and incubated with fluorophore‐conjugated secondary antibodies for 1 h at room temperature. Following this, the slides were stained with Hoechst 33342, and images were captured using a fluorescence microscope (DM6 B; Leica, Wetzlar, Germany). The percentage of NET‐forming neutrophils was defined as the ratio of CitH3 and MPO double‐positive neutrophils to the total neutrophils.

### 4.11 Transwell Assay

Neutrophil transmigration capacity was evaluated using Hanging cell culture inserts (Millicell; Billerica, MA, USA). Briefly, neutrophils (1 × 10^6^/mL) were seeded in the upper chamber, and medium containing fMLP (50 nM) or IL‐8 (20 ng/mL) was placed in the lower chamber. After a 2‐h incubation, migrated cells were fixed with 4% paraformaldehyde, stained with 0.1% crystal violet, and visualized using an optical microscope (DM6 B; Leica).

### 4.12 Seahorse Extracellular Flux Analyzer Assay

Neutrophils (2 × 10^6^/mL) were suspended in Seahorse XF media and seeded into Cell‐Tak‐Coated Seahorse XF24 cell culture plates (Agilent Technologies; Santa Clara, CA, USA). The ECAR was measured using Seahorse XF Glycolysis Stress Test Kit and Seahorse XFe24 Analyzer (Agilent Technologies) according to the manufacturer's instructions.

### 4.13 Statistical Analysis

All quantitative data were expressed as mean ± SEM, representative of at least three independent experiments, and analyzed using GraphPad Prism 8 software (GraphPad Software Inc; San Diego, CA, USA). Statistical comparisons were performed using two‐sided Student's *t*‐test or Tukey's test. Spearman's correlation analysis was used to assess the association between the variables of interest. *p* < 0.05 was considered statistically significant.

## Author Contributions

ZL conceived the study; DK, AL, XX, and HL designed and performed most experiments, conducted the statistical analyses, and drafted the manuscript; LC, ZF, XG, HG, XW, Huiying Lu, Xiaoyu Li, JH, LJ, Haifeng Lian, and XL interpreted the data; LC provided the clinical specimens. All authors have read and approved the final manuscript.

## Funding

This work was funded by grants from the National Natural Science Foundation of China (82370532, 82341219, 82470587, 82270548, and 82270578), the Shanghai Municipal Hospital Development Center (SHDC12022118), the Taishan Scholar Project of Shandong Province (tsqn202306343), and the Qilu Hospital of Shandong University (Qingdao) (QDKY2025RX03).

## Ethics Statement

All experiments were approved by the Institutional Review Board for Clinical Research of the Shanghai Tenth People's Hospital of Tongji University (SHSY‐IEC‐5.0/23K104/P02) and the Institutional Review Board for Animal Research of the Shanghai Tenth People's Hospital of Tongji University (SHDSYY‐2023‐196601). Written informed consent was obtained from all participants.

## Conflicts of Interest

The authors declare no conflicts of interest.

## Supporting information




**Supplementary Figure 1**. Expression levels of bile acid receptors in IBD patients. (A) Relative expression levels of FXR (*NR1H4*), TGR5 (*GPBAR1*), VDR and S1PR2 based on the RNA sequencing data from colorectal tissues of non‐IBD controls and patients with active CD or UC in GEO datasets (GSE75214, GSE165512, GSE117993). (B) Expression of TGR5 (*GPBAR1*), VDR and S1PR2 in the colonic biopsies from healthy controls (HC, n = 20), patients with active CD (A‐CD, n = 20), patients with CD in remission stage (R‐CD, n = 20), patients with active UC (A‐UC, n = 20), and patients with UC in remission stage (R‐UC, n = 20) was analyzed by qRT‐PCR. (C) CD19^+^ B cells, CD56^+^ NK cells, CD4^+^ T cells, CD8^+^ T cells, CD14^+^ monocytes, and CD66b^+^ neutrophils were isolated from peripheral blood of healthy donors (n = 6) and the expression of TGR5 (*GPBAR1*), VDR and S1PR2 was determined by qRT‐PCR. (D) Heatmap showing the transcript levels of *Nr1h4*, *Vdr* and *S1pr2* across different intestinal mucosal immune cell subsets retrieved from a publicly available database. (E) Representative immunofluorescence images of the colonic mucosa stained for DAPI (blue), FXR (green) and MPO (red). Scale bars, 100 µm. (F) Spearman correlation between FXR (*NR1H4*) expression and different immune cell markers in GSE75214 (colorectal tissue, both active and inactive, n = 116), GSE165512 (colorectal tissue, n = 115), and GSE117993 (colorectal tissue, n = 190). Data were expressed as mean ± SEM. Dunnett's test (A) and Tukey's test (B‐C) were used for statistical analysis. *p<0.05; **p<0.01; ***p<0.001; ****p<0.0001; ns, not significant.
**Supplementary Figure 2**. FXR signaling inhibits infiltration and production of proinflammatory mediators of neutrophils in DSS‐induced murine colitis. (A) Eight‐week‐old WT mice were orally given 2% DSS in drinking water from day 3 for 7 days to establish colitis model and the intervention group received daily INT‐747 gavage at a dosage of 5 mg/kg, starting on day 0 (n = 4/group). All mice were sacrificed on day 10. (B) Changes in body weight during a 10‐day modeling period. (C) The gross morphology and length of the colon were assessed on day 10. (D) The mRNA expression of various inflammatory mediators in the colonic tissues. (E) Representative images of the colonic sections after hematoxylin and eosin (H&E) staining. Scale bars, 300 µm. Pathological scores are shown in the bar chart. (F) Gating strategy used for flow cytometric analysis of lamina propria‐infiltrating immune cells. (G) Flow cytometric analysis of lamina propria‐infiltrating neutrophils. LP, lamina propria. (H‐I) Quantifications of lamina propria‐infiltrating CD4^+^ T cells (H) and macrophages (I) were performed by flow cytometry. (J) Representative immunofluorescence staining of specific markers for neutrophil extracellular traps (NETs) in the colonic sections. Scale bars, 50 µm. Quantification of NET‐forming neutrophils is shown in the bar chart. Data were expressed as mean ± SEM. Tukey's test was used for statistical analysis. *p<0.05; **p<0.01; ***p<0.001; ****p<0.0001; ns, not significant.
**Supplementary Figure 3**. FXR signaling inhibits the proinflammatory phenotypes in murine colitis models. (A) Relative mRNA expression of *Il1b*, *Il17a*, *Ifng*, and *Cxcl1* in the colonic tissues described in Figure 2D (n = 5/group). (B) Flow cytometric quantification of lamina propria‐infiltrating neutrophils described in Figure 2F (n = 5/group). LP, lamina propria. (C‐D) Quantifications of CD4^+^ T cells (C) and macrophages (D) infiltration within the lamina propria, as described in Figure 2F, were performed by flow cytometry (n = 5/group). (E) Relative mRNA expression of *Il1b*, *Il17a*, *Ifng*, and *Cxcl1* in the colonic tissues described in Figure 2K (n = 5/group). (F) Flow cytometric quantification of lamina propria‐infiltrating neutrophils described in Figure 2M (n = 5/group). (G‐H) Quantifications of CD4^+^ T cells (G) and macrophages (H) infiltration within the lamina propria, as described in Figure 2M, were performed by flow cytometry (n = 5/group). (I) Relative mRNA expression of *Il1b*, *Il17a*, *Ifng*, and *Cxcl1* in the colonic tissues described in Figure 3D (n = 5/group). (J) Flow cytometric quantification of lamina propria‐infiltrating neutrophils described in Figure 3F (n = 5/group). (K‐L) Quantifications of CD4^+^ T cells (K) and macrophages (L) infiltration within the lamina propria, as described in Figure 3F, were performed by flow cytometry (n = 5/group). Data were expressed as mean ± SEM. Tukey's test (A‐B) and Student's *t*‐test (C‐L) were used for statistical analysis. *p<0.05; **p<0.01; ***p<0.001; ****p<0.0001; ns, not significant.
**Supplementary Figure 4**. *Fxr* ^−/−^ neutrophils display enhanced proinflammatory functions. Bone marrow (BM)‐derived neutrophils (n = 3/group) were harvested from WT and *Fxr* ^−/−^ mice for RNA sequencing analysis. (A) Volcano plot of differentially expressed genes between two groups. (B) GO analysis of differentially expressed genes. (C) KEGG pathway analysis of differentially expressed genes. (D) BM‐derived neutrophils (3 × 10^6^/mL) were isolated from WT and *Fxr* ^−/−^ mice and treated with or without LPS (300 ng/mL) for 3 h. Expression levels of proinflammatory mediators were detected by qRT‐PCR (n = 6/group). (E‐F) The levels of ROS (E) and MPO (F) produced by BM‐derived neutrophils (1 × 10^6^/mL) following 3 h of LPS (300 ng/mL) treatment were measured by Amplex Red Hydrogen Peroxide/Peroxidase Assay Kit according to the manufacturer's instructions (n = 5/group). Data were expressed as mean ± SEM. Tukey's test (D‐F) was used for statistical analysis. *p<0.05; **p<0.01; ***p<0.001; ****p<0.0001; ns, not significant.
**Supplementary Figure 5**. FXR regulates neutrophil functions through the mTORC1‐glycolysis pathway. (A) Gene set enrichment analysis (GSEA) of the RNA sequencing data described in Figure 4. (B) Phospho‐flow analysis of the mTORC2 substrate Akt phosphorylated at Ser473 (p‐Akt, S473), in BM‐derived neutrophils (3 × 10^6^/mL) under 3 h of LPS (300 ng/mL) treatment (n = 5/group). MFI, mean fluorescence intensity. (C) BM‐derived neutrophils (3 × 10^6^/mL) were isolated from WT and *Fxr* ^−/−^ mice and treated with or without LPS (300 ng/mL) for 3 h. Expression levels of glycolysis‐related genes were detected by qRT‐PCR (n = 6/group). (D) Relative glycolytic capacity detected by Seahorse described in Figure 5F. (E‐F) BM‐derived neutrophils (1 × 10^6^/mL) were treated with LPS (300 ng/mL) in the presence or absence of rapamycin (2 µM) or 2‐Deoxy‐D‐glucose (2‐DG, 5 mM) for 3 h. The levels of ROS (E) and MPO (F) were measured by Amplex Red Hydrogen Peroxide/Peroxidase Assay Kit according to the manufacturer's instructions (n = 5/group). (G‐I) BM‐derived neutrophils (3 × 10^6^/mL) were treated with LPS (300 ng/mL) in the presence or absence of rapamycin (2 µM) for 3 h. (G) Expression levels of glycolysis‐related genes were detected by qRT‐PCR (n = 6/group). (H) The glucose uptake capacity was measured by flow cytometry using 2‐NBDG (n = 5/group). (I) The extracellular acidification rate (ECAR) of neutrophils was detected by Seahorse (n = 3/group). Glu, glucose; Oli, oligomycin. Data were expressed as mean ± SEM. Tukey's test was used for statistical analysis. *p<0.05; **p<0.01; ***p<0.001; ****p<0.0001; ns, not significant.
**Supplementary Figure 6**. The mTORC1‐glycolysis signaling facilitates neutrophil hyperactivation in DSS‐induced colitis in *Fxr* ^−/−^ mice. (A) Eight‐week‐old WT and *Fxr* ^−/−^ mice were administered 2% DSS in drinking water for 7 days, followed by 3 days of regular water, to induce colitis. In the mTORC1 inhibition group, mice received daily oral gavage of rapamycin (1.5 mg/kg). In the glycolysis inhibition group, mice were treated with 2‐DG (500 mg/kg) via intraperitoneal injection daily. All mice were sacrificed on day 10 (n = 5/group). (B) Changes in body weight over a 10‐day modeling period. (C) The gross morphology and length of colon on day 10. (D) Expression of various inflammatory mediators in the colonic tissues was analyzed by qRT‐PCR. (E) H&E staining and histopathological scores were applied to assess the severity of colitis. Scale bars, 200 µm. (F) Flow cytometric analysis of lamina propria‐infiltrating neutrophils. LP, lamina propria. (G) Representative immunofluorescence staining of specific markers for NETs in the colonic sections. Scale bars, 30 µm. Quantification of NET‐forming neutrophils is shown in the bar chart. Data were expressed as mean ± SEM. Tukey's test was used for statistical analysis. *p<0.05; **p<0.01; ***p<0.001; ****p<0.0001; ns, not significant.
**Supplementary Figure 7**. Differentially expressed gene profiles and pathway analysis. Peripheral blood neutrophils (3 × 10^6^/mL) isolated from healthy donors (HC, n = 3), patients with active CD (n = 3), and patients with active UC (n = 3) were treated with or without INT‐747 (40 µM) for 3 h. The total RNA was extracted for RNA sequencing analysis. (A) Volcano plot of differentially expressed genes. (B‐C) GO analysis of differentially expressed genes in the indicated comparisons. (D) Gene set variation analysis (GSVA) in the indicated comparisons. NES, normalized enrichment score. (E) Heatmap showing expression of genes associated with neutrophil functions.
**Supplementary Figure 8**. FXR signaling inhibits neutrophil production of proinflammatory mediators. Peripheral blood (PB) neutrophils (3 × 10^6^/mL) isolated from healthy donors (HC, n = 6), patients with active CD (n = 6), and patients with active UC (n = 6) were stimulated with LPS (300 ng/mL) in the presence or absence of INT‐747 (40 µM) for 3 h. Cells were collected to determine mRNA expression by qRT‐PCR. For ELISA analysis, PB neutrophils (2 × 10^6^/mL) were stimulated with LPS (300 ng/mL) in the presence or absence of INT‐747 (40 µM) for 24 h, and the culture supernatants were then collected for detection. (A) The mRNA expression levels of *TNF*, *IL1B*, and *IL6*. (B) The protein levels of TNF‐α, IL‐1β, and IL‐6 in culture supernatants. (C) The mRNA expression levels of *S100A8*, *S100A9*, and *LCN2*. (D) The protein levels of S100A8/9 and LCN2 in culture supernatants. (E‐F) The mRNA expression level (E) and protein level (F) of IL‐8. (G‐H) The levels of ROS (G) and MPO (H) produced by PB neutrophils (1 × 10^6^/mL) following 3 h of LPS (300 ng/mL) treatment were measured by Amplex Red Hydrogen Peroxide/Peroxidase Assay Kit according to the manufacturer's instructions (n = 6/group). Data were expressed as mean ± SEM. Tukey's test was used for statistical analysis. *p<0.05; **p<0.01; ***p<0.001; ****p<0.0001; ns, not significant.
**Supplementary Figure 9**. FXR signaling downregulates glycolysis‐related genes in neutrophils. (A) PB neutrophils (3 × 10^6^/mL) isolated from healthy donors (HC, n = 6), patients with active CD (n = 6), and patients with active UC (n = 6) were stimulated with LPS (300 ng/mL) in the presence or absence of INT‐747 (40 µM) for 3 h. The expression levels of glycolysis‐related genes were measured by qRT‐PCR. (B) Bar chart of the relative glycolytic capacity described in Figure 6G. Data were expressed as mean ± SEM. Tukey's test was used for statistical analysis. *p<0.05; **p<0.01; ***p<0.001; ****p<0.0001; ns, not significant.
**Supplementary Table 1**. Reagents used in this study.
**Supplementary Table 2**. Characteristics of IBD patients and healthy donors.
**Supplementary Table 3**. Primers used for qRT‐PCR analysis.

## Data Availability

All data relevant to the study are included in the article or uploaded as Supporting Information or from the corresponding author upon reasonable request. The RNA sequencing data have been submitted to the National Genomics Data Center of China National Center for Bioinformation with the accession numbers CRA031173 and HRA013809.
