# Supplementary material for: Dysregulation of Farnesoid X Receptor on Neutrophil Homeostasis Exacerbates Intestinal Inflammation via the mTORC1‐Glycolysis Signaling Pathway
Source: MedComm (2020). 2026 Feb 8;7(2):e70637. doi: 10.1002/mco2.70637 (PMC12883035; doi:10.1002/mco2.70637)
Supplement: Supplementary file 1 — Supplementary Figure 1. Expression levels of bile acid receptors in IBD patients. (A) Relative expression levels of FXR (NR1H4), TGR5 (GPBAR1), VDR and S1PR2 based on the RNA sequencing data from colorectal tissues of non‐IBD controls and patients with active CD or UC in GEO datasets (GSE75214, GSE165512, GSE117993). (B) Expression of TGR5 (GPBAR1), VDR and S1PR2 in the colonic biopsies from healthy controls (HC, n = 20), patients with active CD (A‐CD, n = 20), patients with CD in remission stage (R‐CD, n = 20), patients with active UC (A‐UC, n = 20), and patients with UC in remission stage (R‐UC, n = 20) was analyzed by qRT‐PCR. (C) CD19+ B cells, CD56+ NK cells, CD4+ T cells, CD8+ T cells, CD14+ monocytes, and CD66b+ neutrophils were isolated from peripheral blood of healthy donors (n = 6) and the expression of TGR5 (GPBAR1), VDR and S1PR2 was determined by qRT‐PCR. (D) Heatmap showing the transcript levels of Nr1h4, Vdr and S1pr2 across different intestinal mucosal immune cell subsets retrieved from a publicly available database. (E) Representative immunofluorescence images of the colonic mucosa stained for DAPI (blue), FXR (green) and MPO (red). Scale bars, 100 µm. (F) Spearman correlation between FXR (NR1H4) expression and different immune cell markers in GSE75214 (colorectal tissue, both active and inactive, n = 116), GSE165512 (colorectal tissue, n = 115), and GSE117993 (colorectal tissue, n = 190). Data were expressed as mean ± SEM. Dunnett's test (A) and Tukey's test (B‐C) were used for statistical analysis. *p<0.05; **p<0.01; ***p<0.001; ****p<0.0001; ns, not significant. Supplementary Figure 2. FXR signaling inhibits infiltration and production of proinflammatory mediators of neutrophils in DSS‐induced murine colitis. (A) Eight‐week‐old WT mice were orally given 2% DSS in drinking water from day 3 for 7 days to establish colitis model and the intervention group received daily INT‐747 gavage at a dosage of 5 mg/kg, starting on day 0 (n = 4/group). All mice [file MCO2-7-e70637-s001.docx]

Supplementary Materials for

**Dysregulation of farnesoid X receptor on neutrophil homeostasis exacerbates intestinal inflammation via the mTORC1-glycolysis signaling pathway**

Dengfeng Kang *et al*.

*Corresponding author: Prof. Dr. Zhanju Liu, E-mail: liuzhanju88@126.com

**This file includes:**

Supplementary Methods

Supplementary Figures 1-9

Supplementary Tables 1-3

**Supplementary methods**

**1. Quantitative real-time polymerase chain reaction (qRT-PCR)**

Total RNA was extracted using the TRIzol reagent and used as the template for complementary DNA synthesis. The reverse transcription was carried out following the manufacturer’s protocol with a 5 × All-in-one RT MasterMix Kit (Applied Biological Materials Inc.; Richmond, BC, Canada). qRT-PCR was performed using a TB Green Premix Ex Taq PCR Kit (TaKaRa; Dalian, China) based on the manufacturer’s instructions in the 7900HT Fast Real-Time PCR system (Applied Biosystems; Carlsbad, CA, USA). The primer sequences are detailed in Table S3.

**2. Measurement of extracellular ROS and MPO**

Extracellular ROS and MPO levels of neutrophils were quantified using the Amplex Red Hydrogen Peroxide/Peroxidase Assay Kit (Invitrogen; Eugene, OR, USA) according to the manufacturer’s instructions. Briefly, neutrophils (1 × 10^6^/mL) were seeded in 96-well plates and incubated with the Amplex Red working solution for 1 hour at 37℃. Absorbance was subsequently measured at 560 nm using a microplate spectrophotometer (BioTek; Winooski, VT, USA).

**3. Enzyme-linked immunosorbent assay (ELISA)**

The levels of proinflammatory mediators in neutrophil culture supernatants were quantified using ELISA kits (BioLegend; San Diego, CA, USA) according to the manufacturer’s instructions. Briefly, the samples were added to pre-coated 96-well plates, followed by incubation with detection antibody and avidin-HRP. After addition of TMB substrate, the absorbance was measured at 450 nm using a microplate spectrophotometer (BioTek; Winuschi, VT, USA).

**Supplementary figures and figure legends**


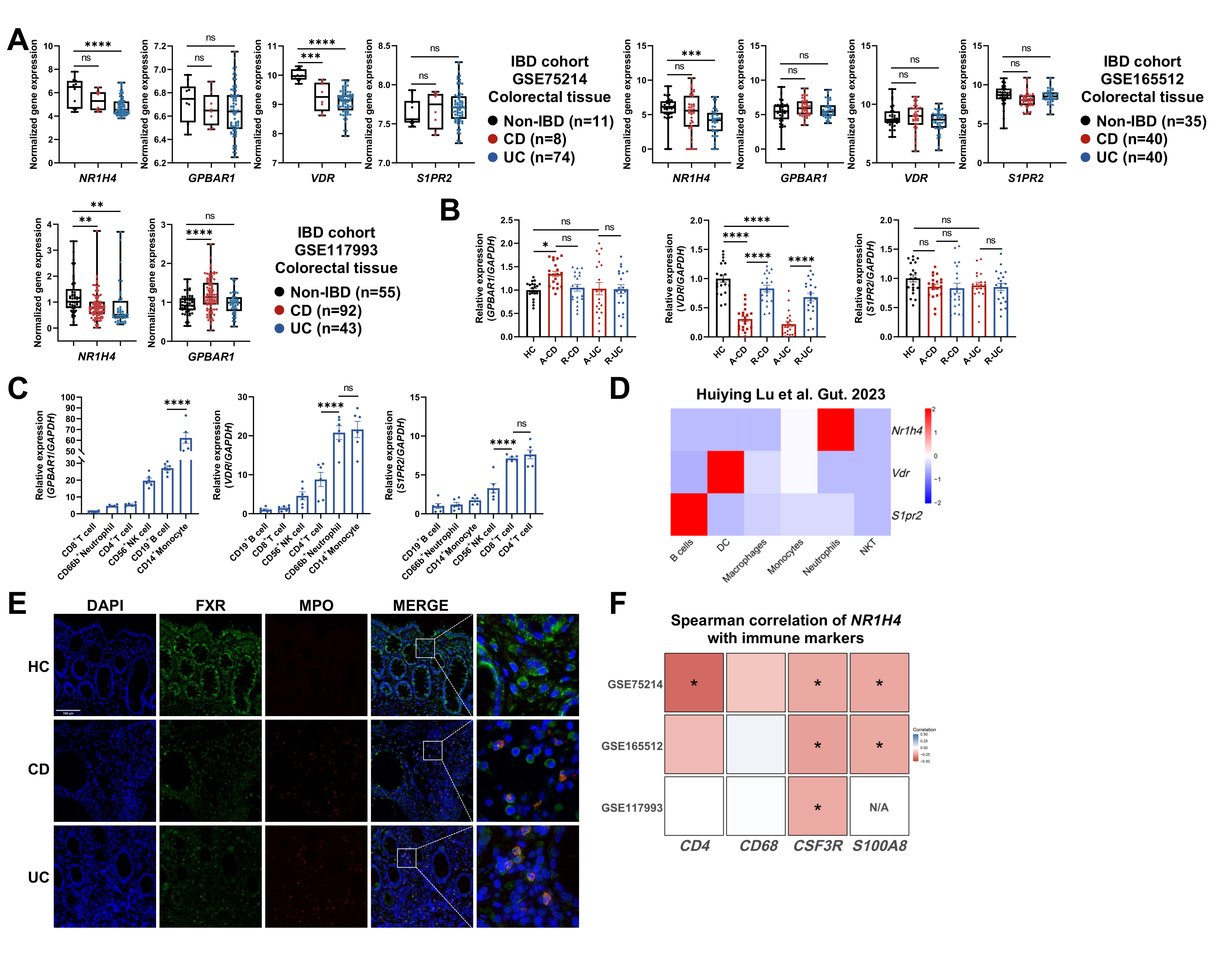


**Supplementary Figure 1.** **Expression levels of bile acid receptors in IBD patients.** (**A**) Relative expression levels of FXR (*NR1H4*), TGR5 (*GPBAR1*), VDR and S1PR2 based on the RNA sequencing data from colorectal tissues of non-IBD controls and patients with active CD or UC in GEO datasets (GSE75214, GSE165512, GSE117993). (**B**) Expression of TGR5 (*GPBAR1*), VDR and S1PR2 in the colonic biopsies from healthy controls (HC, n=20), patients with active CD (A-CD, n=20), patients with CD in remission stage (R-CD, n=20), patients with active UC (A-UC, n=20), and patients with UC in remission stage (R-UC, n=20) was analyzed by qRT-PCR. (**C**) CD19^+^ B cells, CD56^+^ NK cells, CD4^+^ T cells, CD8^+^ T cells, CD14^+^ monocytes, and CD66b^+^ neutrophils were isolated from peripheral blood of healthy donors (n=6) and the expression of TGR5 (*GPBAR1*), VDR and S1PR2 was determined by qRT-PCR. (**D**) Heatmap showing the transcript levels of *Nr1h4*, *Vdr* and *S1pr2* across different intestinal mucosal immune cell subsets retrieved from a publicly available database. (**E**) Representative immunofluorescence images of the colonic mucosa stained for DAPI (blue), FXR (green) and MPO (red). Scale bars, 100 μm. (**F**) Spearman correlation between FXR (*NR1H4*) expression and different immune cell markers in GSE75214 (colorectal tissue, both active and inactive, n=116), GSE165512 (colorectal tissue, n=115), and GSE117993 (colorectal tissue, n=190). Data were expressed as mean ± SEM. Dunnett’s test (A) and Tukey’s test (B-C) were used for statistical analysis. **p*<0.05; ***p*<0.01; ****p*<0.001; *****p*<0.0001; ns, not significant.


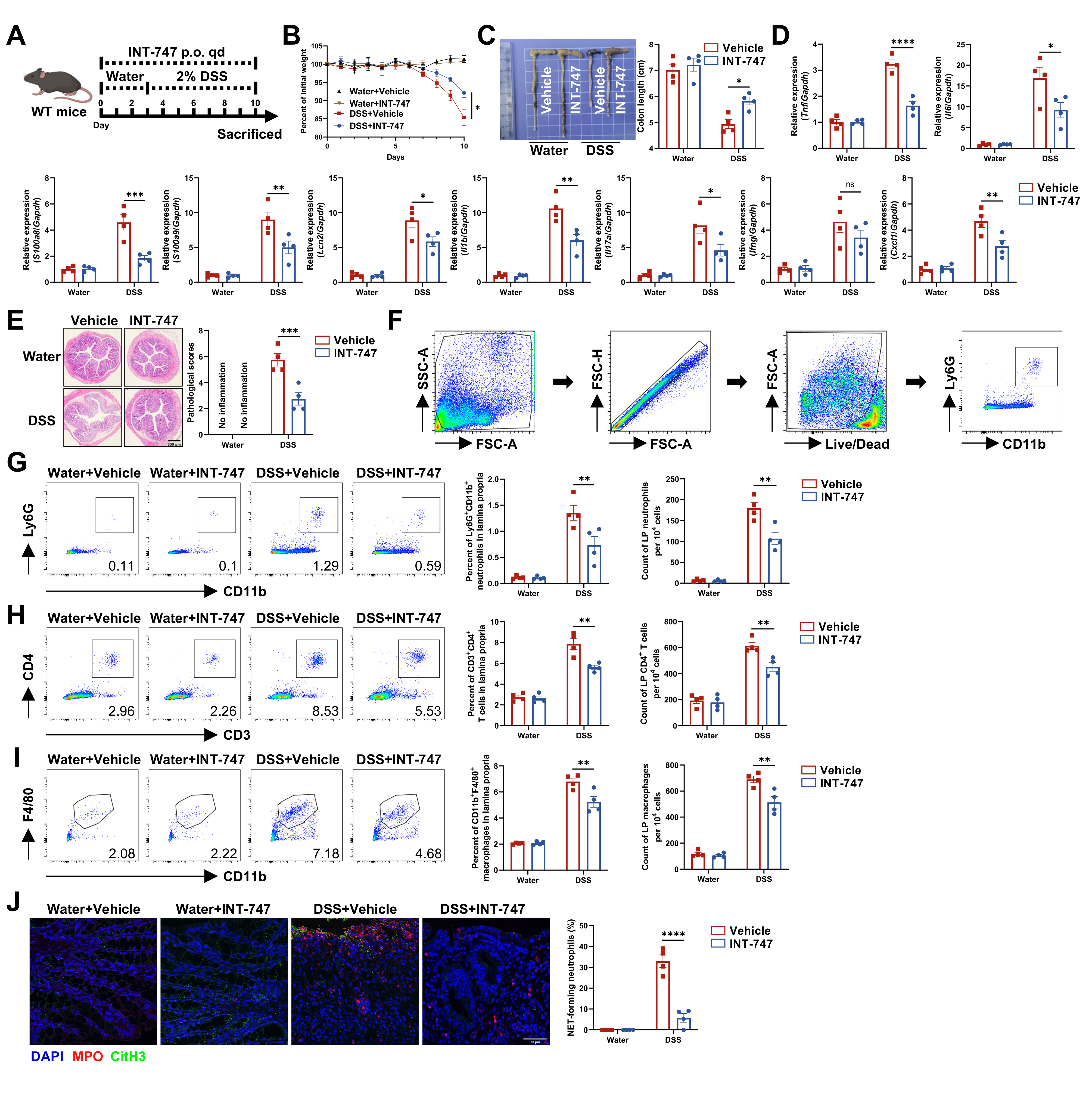


**Supplementary Figure 2.** **FXR signaling inhibits infiltration and production of proinflammatory mediators of neutrophils in DSS-induced murine colitis.** (**A**) Eight-week-old WT mice were orally given 2% DSS in drinking water from day 3 for 7 days to establish colitis model and the intervention group received daily INT-747 gavage at a dosage of 5 mg/kg, starting on day 0 (n=4/group). All mice were sacrificed on day 10. (**B**) Changes in body weight during a 10-day modeling period. (**C**) The gross morphology and length of the colon were assessed on day 10. (**D**) The mRNA expression of various inflammatory mediators in the colonic tissues. (**E**) Representative images of the colonic sections after hematoxylin and eosin (H&E) staining. Scale bars, 300 μm. Pathological scores are shown in the bar chart. (**F**) Gating strategy used for flow cytometric analysis of lamina propria-infiltrating immune cells. (**G**) Flow cytometric analysis of lamina propria-infiltrating neutrophils. LP, lamina propria. (**H-I**) Quantifications of lamina propria-infiltrating CD4^+^ T cells (H) and macrophages (I) were performed by flow cytometry. (**J**) Representative immunofluorescence staining of specific markers for neutrophil extracellular traps (NETs) in the colonic sections. Scale bars, 50 μm. Quantification of NET-forming neutrophils is shown in the bar chart. Data were expressed as mean ± SEM. Tukey’s test was used for statistical analysis. **p*<0.05; ***p*<0.01; ****p*<0.001; *****p*<0.0001; ns, not significant.


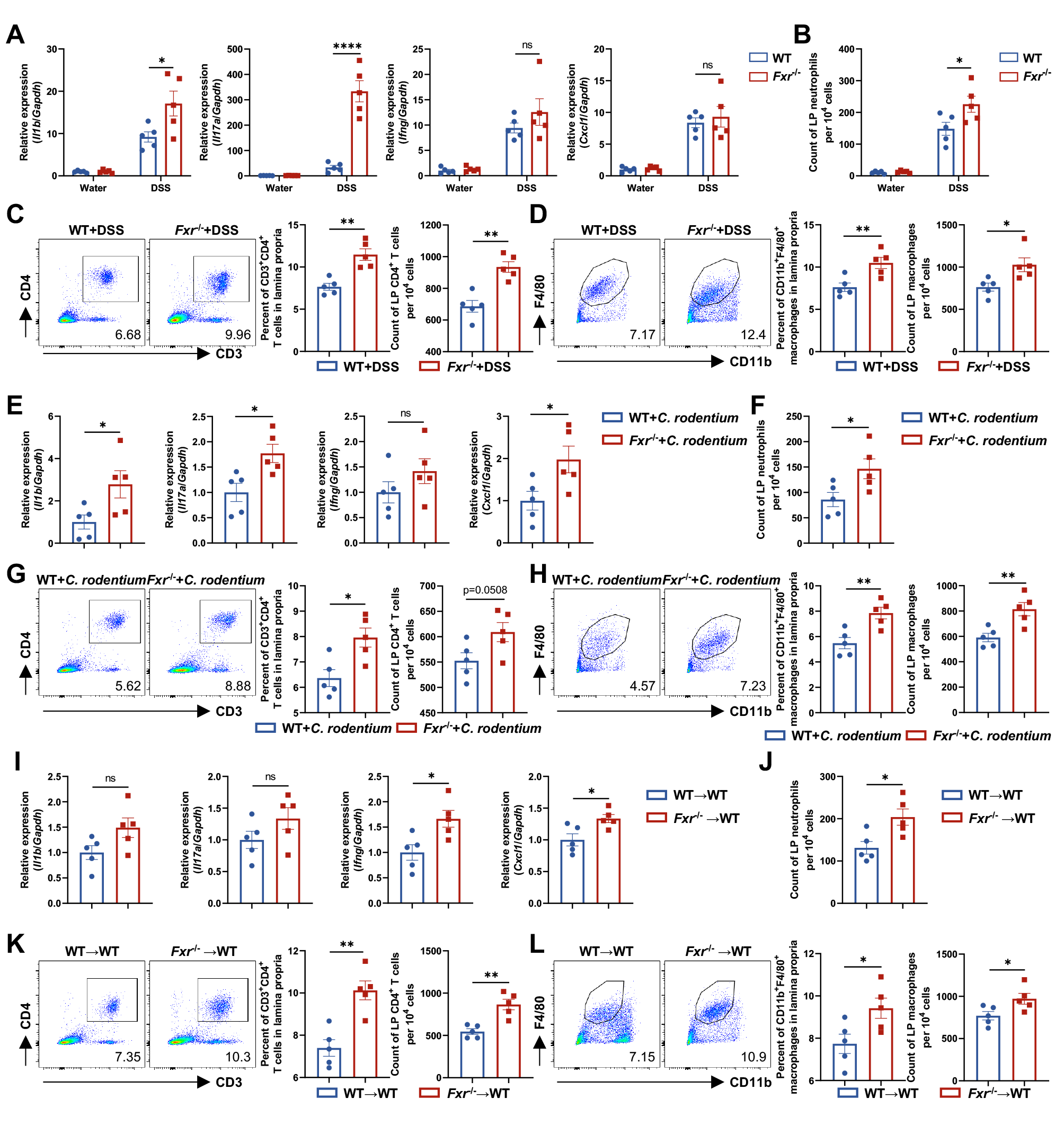


**Supplementary Figure 3.** **FXR signaling inhibits the proinflammatory phenotypes in murine colitis models.** (**A**) Relative mRNA expression of *Il1b*, *Il17a*, *Ifng*, and *Cxcl1* in the colonic tissues described in Figure 2D (n=5/group). (**B**) Flow cytometric quantification of lamina propria-infiltrating neutrophils described in Figure 2F (n=5/group). LP, lamina propria. (**C-D**) Quantifications of CD4^+^ T cells (C) and macrophages (D) infiltration within the lamina propria, as described in Figure 2F, were performed by flow cytometry (n=5/group). (**E**) Relative mRNA expression of *Il1b*, *Il17a*, *Ifng*, and *Cxcl1* in the colonic tissues described in Figure 2K (n=5/group). (**F**) Flow cytometric quantification of lamina propria-infiltrating neutrophils described in Figure 2M (n=5/group). (**G-H**) Quantifications of CD4^+^ T cells (G) and macrophages (H) infiltration within the lamina propria, as described in Figure 2M, were performed by flow cytometry (n=5/group). (**I**) Relative mRNA expression of *Il1b*, *Il17a*, *Ifng*, and *Cxcl1* in the colonic tissues described in Figure 3D (n=5/group). (**J**) Flow cytometric quantification of lamina propria-infiltrating neutrophils described in Figure 3F (n=5/group). (**K-L**) Quantifications of CD4^+^ T cells (K) and macrophages (L) infiltration within the lamina propria, as described in Figure 3F, were performed by flow cytometry (n=5/group). Data were expressed as mean ± SEM. Tukey’s test (A-B) and Student’s *t*-test (C-L) were used for statistical analysis. **p*<0.05; ***p*<0.01; ****p*<0.001; *****p*<0.0001; ns, not significant.


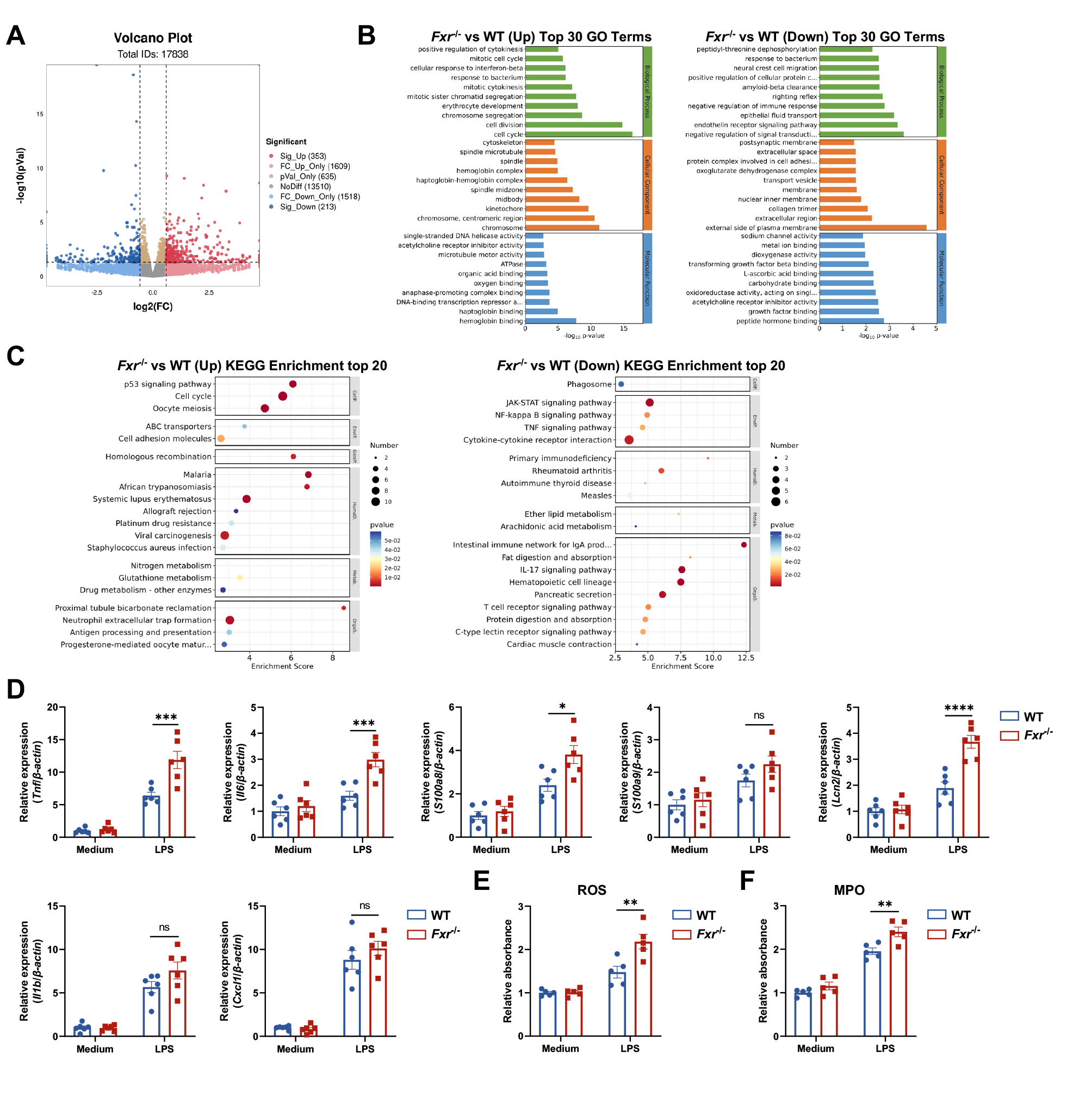


**Supplementary Figure 4.** ***Fxr*^-/-^ neutrophils display enhanced proinflammatory functions.** Bone marrow (BM)-derived neutrophils (n=3/group) were harvested from WT and *Fxr*^-/-^ mice for RNA sequencing analysis. (**A**) Volcano plot of differentially expressed genes between two groups. (**B**) GO analysis of differentially expressed genes. (**C**) KEGG pathway analysis of differentially expressed genes. (**D**) BM-derived neutrophils (3 × 10^6^/mL) were isolated from WT and *Fxr*^-/-^ mice and treated with or without LPS (300 ng/mL) for 3 hours. Expression levels of proinflammatory mediators were detected by qRT-PCR (n=6/group). (**E-F**) The levels of ROS (E) and MPO (F) produced by BM-derived neutrophils (1 × 10^6^/mL) following 3 hours of LPS (300 ng/mL) treatment were measured by Amplex Red Hydrogen Peroxide/Peroxidase Assay Kit according to the manufacturer’s instructions (n=5/group). Data were expressed as mean ± SEM. Tukey’s test (D-F) was used for statistical analysis. **p*<0.05; ***p*<0.01; ****p*<0.001; *****p*<0.0001; ns, not significant.


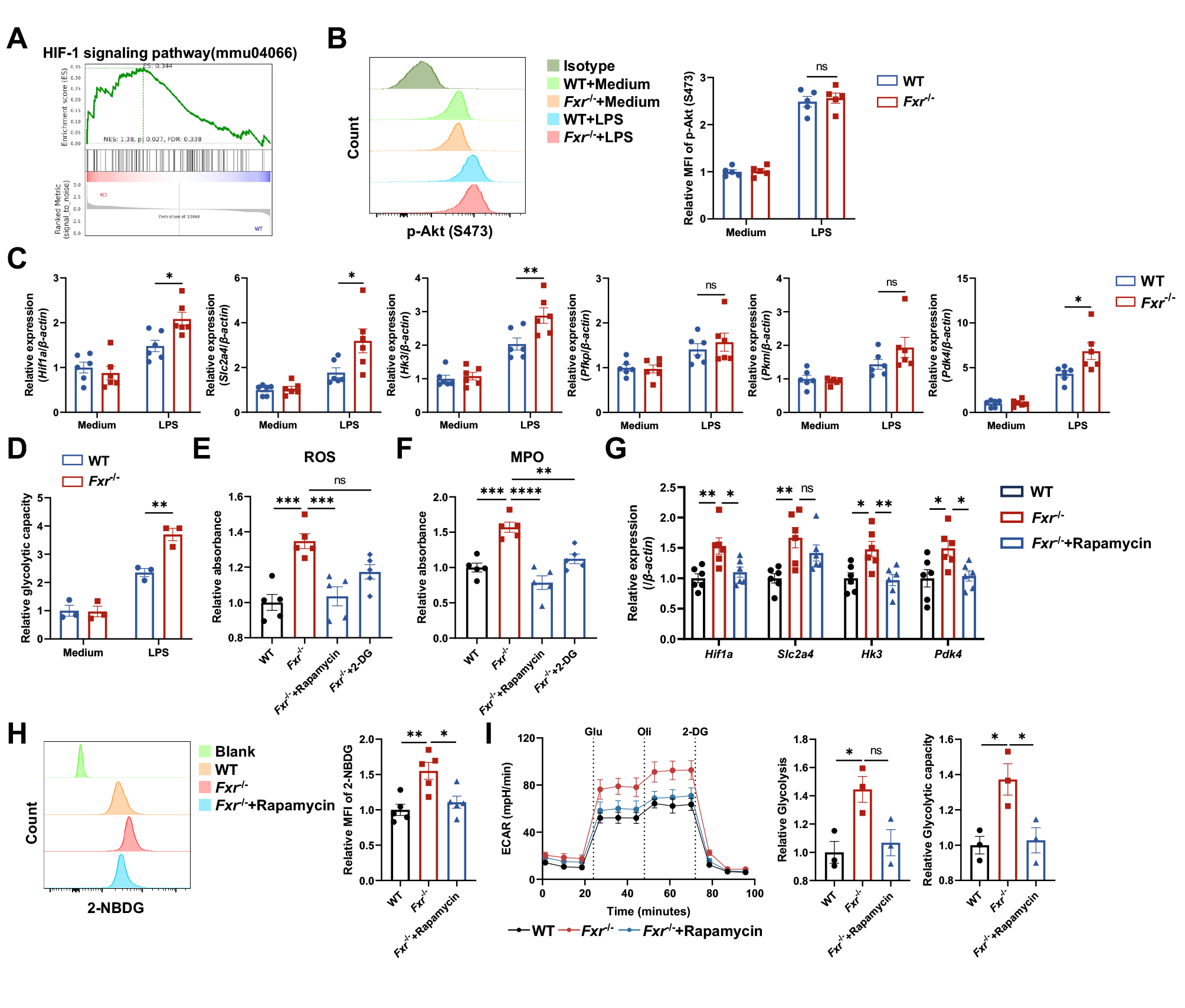


**Supplementary Figure 5.** **FXR regulates neutrophil functions through the mTORC1-glycolysis pathway.** (**A**) Gene set enrichment analysis (GSEA) of the RNA sequencing data described in Figure 4. (**B**) Phospho-flow analysis of the mTORC2 substrate Akt phosphorylated at Ser473 (p-Akt, S473), in BM-derived neutrophils (3 × 10^6^/mL) under 3 hours of LPS (300 ng/mL) treatment (n=5/group). MFI, mean fluorescence intensity. (**C**) BM-derived neutrophils (3 × 10^6^/mL) were isolated from WT and *Fxr*^-/-^ mice and treated with or without LPS (300 ng/mL) for 3 hours. Expression levels of glycolysis-related genes were detected by qRT-PCR (n=6/group). (**D**) Relative glycolytic capacity detected by Seahorse described in Figure 5F. (**E-F**) BM-derived neutrophils (1 × 10^6^/mL) were treated with LPS (300 ng/mL) in the presence or absence of rapamycin (2 μM) or 2-Deoxy-D-glucose (2-DG, 5 mM) for 3 hours. The levels of ROS (E) and MPO (F) were measured by Amplex Red Hydrogen Peroxide/Peroxidase Assay Kit according to the manufacturer’s instructions (n=5/group). (**G-I**) BM-derived neutrophils (3 × 10^6^/mL) were treated with LPS (300 ng/mL) in the presence or absence of rapamycin (2 μM) for 3 hours. (G) Expression levels of glycolysis-related genes were detected by qRT-PCR (n=6/group). (H) The glucose uptake capacity was measured by flow cytometry using 2-NBDG (n=5/group). (I) The extracellular acidification rate (ECAR) of neutrophils was detected by Seahorse (n=3/group). Glu, glucose; Oli, oligomycin. Data were expressed as mean ± SEM. Tukey’s test was used for statistical analysis. **p*<0.05; ***p*<0.01; ****p*<0.001; *****p*<0.0001; ns, not significant.


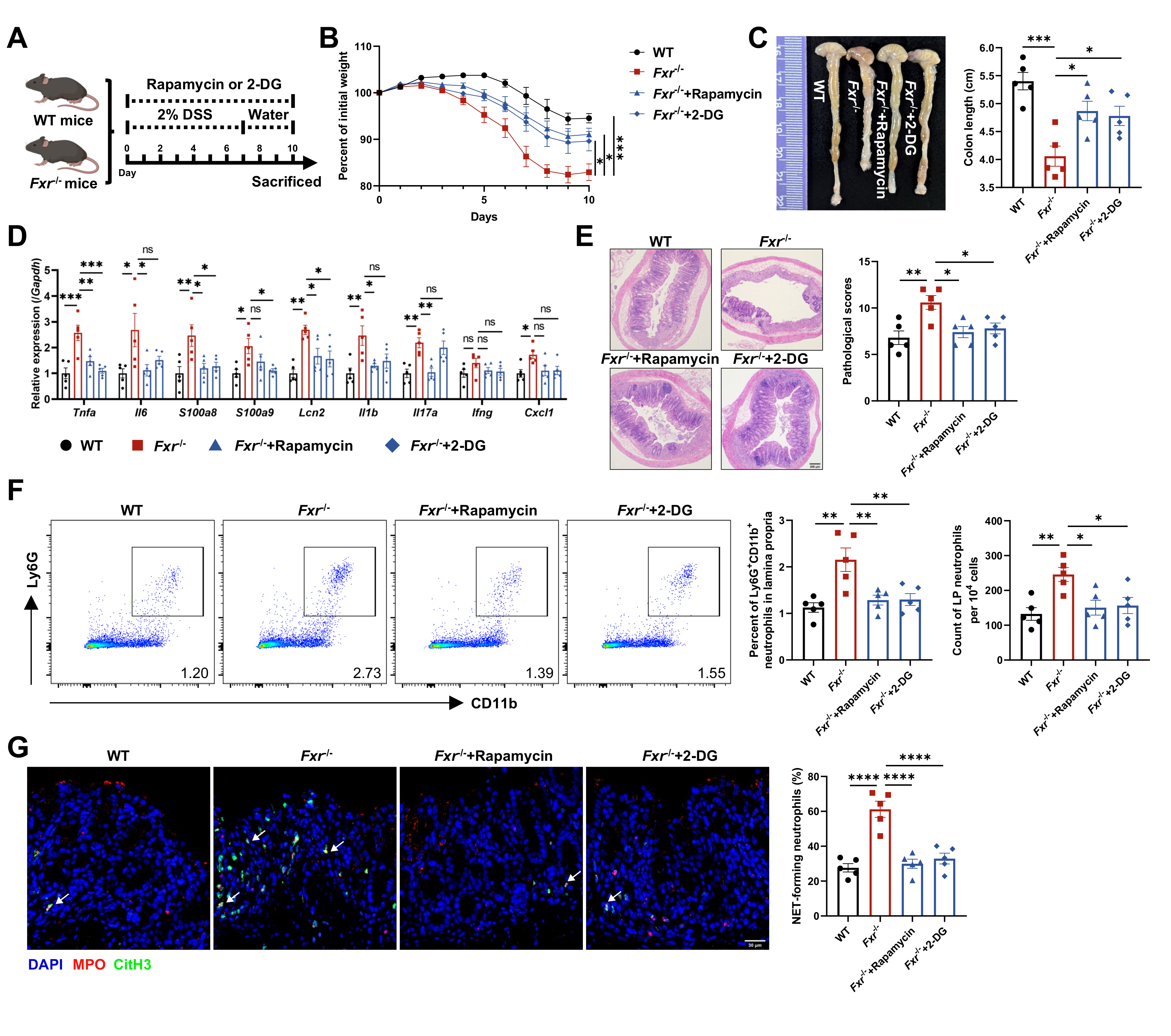


**Supplementary Figure 6.** **The mTORC1-glycolysis signaling facilitates neutrophil hyperactivation in DSS-induced colitis in *Fxr*^-/-^ mice.** (**A**) Eight-week-old WT and *Fxr*^-/-^ mice were administered 2% DSS in drinking water for 7 days, followed by 3 days of regular water, to induce colitis. In the mTORC1 inhibition group, mice received daily oral gavage of rapamycin (1.5mg/kg). In the glycolysis inhibition group, mice were treated with 2-DG (500mg/kg) via intraperitoneal injection daily. All mice were sacrificed on day 10 (n=5/group). (**B**) Changes in body weight over a 10-day modeling period. (**C**) The gross morphology and length of colon on day 10. (**D**) Expression of various inflammatory mediators in the colonic tissues was analyzed by qRT-PCR. (**E**) H&E staining and histopathological scores were applied to assess the severity of colitis. Scale bars, 200 μm. (**F**) Flow cytometric analysis of lamina propria-infiltrating neutrophils. LP, lamina propria. (**G**) Representative immunofluorescence staining of specific markers for NETs in the colonic sections. Scale bars, 30 μm. Quantification of NET-forming neutrophils is shown in the bar chart. Data were expressed as mean ± SEM. Tukey’s test was used for statistical analysis. **p*<0.05; ***p*<0.01; ****p*<0.001; *****p*<0.0001; ns, not significant.


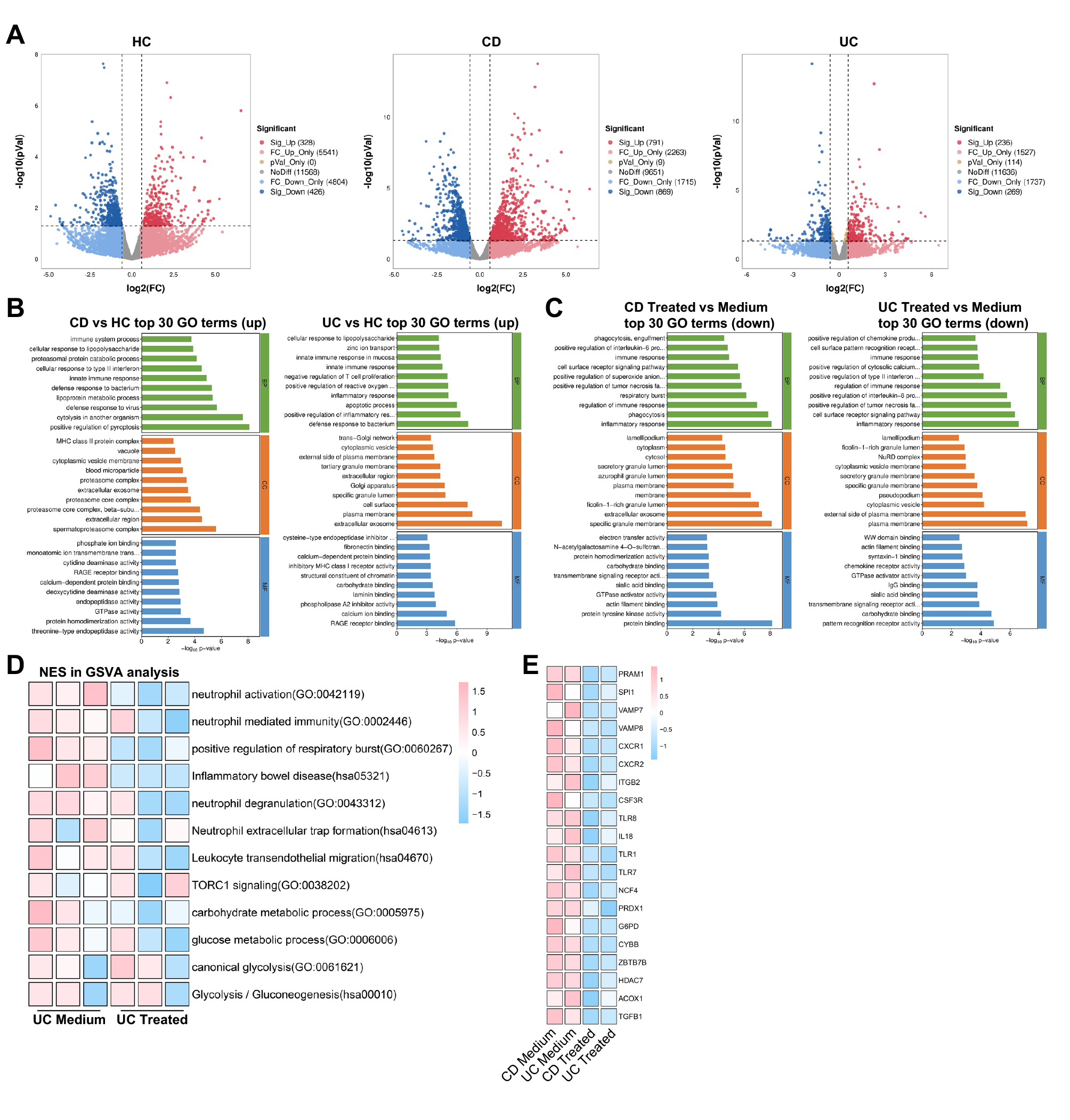


**Supplementary Figure 7.** **Differentially expressed gene profiles and pathway analysis.** Peripheral blood neutrophils (3 × 10^6^/mL) isolated from healthy donors (HC, n=3), patients with active CD (n=3), and patients with active UC (n=3) were treated with or without INT-747 (40 μM) for 3 hours. The total RNA was extracted for RNA sequencing analysis. (**A**) Volcano plot of differentially expressed genes. (**B-C**) GO analysis of differentially expressed genes in the indicated comparisons. (**D**) Gene set variation analysis (GSVA) in the indicated comparisons. NES, normalized enrichment score. (**E**) Heatmap showing expression of genes associated with neutrophil functions.


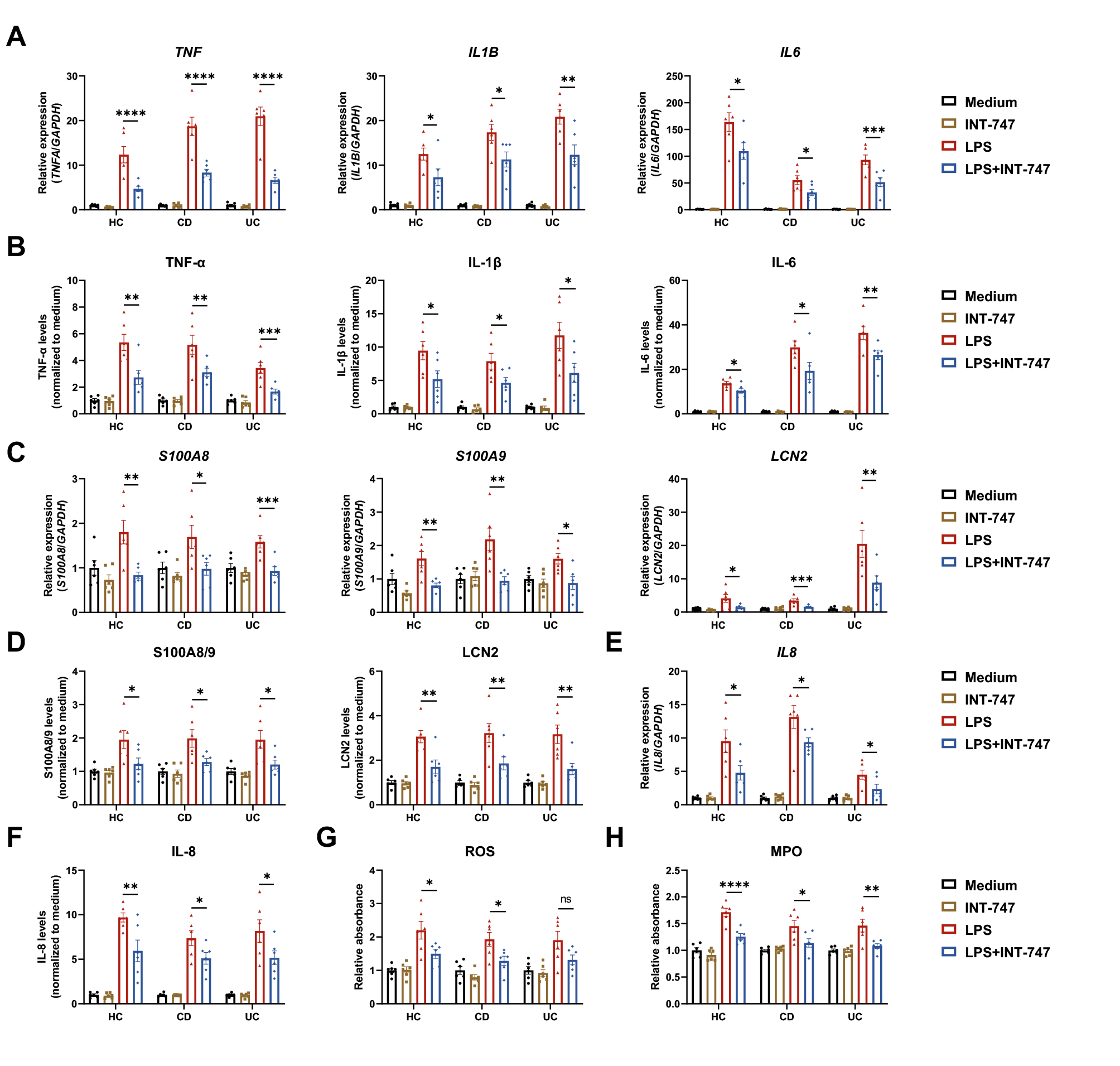


**Supplementary Figure 8.** **FXR signaling inhibits neutrophil production of proinflammatory mediators.** Peripheral blood (PB) neutrophils (3 × 10^6^/mL) isolated from healthy donors (HC, n=6), patients with active CD (n=6), and patients with active UC (n=6) were stimulated with LPS (300 ng/mL) in the presence or absence of INT-747 (40 μM) for 3 hours. Cells were collected to determine mRNA expression by qRT-PCR. For ELISA analysis, PB neutrophils (2 × 10^6^/mL) were stimulated with LPS (300 ng/mL) in the presence or absence of INT-747 (40 μM) for 24 hours, and the culture supernatants were then collected for detection. (**A**) The mRNA expression levels of *TNF*, *IL1B*, and *IL6*. (**B**) The protein levels of TNF-α, IL-1β, and IL-6 in culture supernatants. (**C**) The mRNA expression levels of *S100A8*, *S100A9*, and *LCN2*. (**D**) The protein levels of S100A8/9 and LCN2 in culture supernatants. (**E**-**F**) The mRNA expression level (E) and protein level (F) of IL-8. (**G-H**) The levels of ROS (G) and MPO (H) produced by PB neutrophils (1 × 10^6^/mL) following 3 hours of LPS (300 ng/mL) treatment were measured by Amplex Red Hydrogen Peroxide/Peroxidase Assay Kit according to the manufacturer’s instructions (n=6/group). Data were expressed as mean ± SEM. Tukey’s test was used for statistical analysis. **p*<0.05; ***p*<0.01; ****p*<0.001; *****p*<0.0001; ns, not significant.


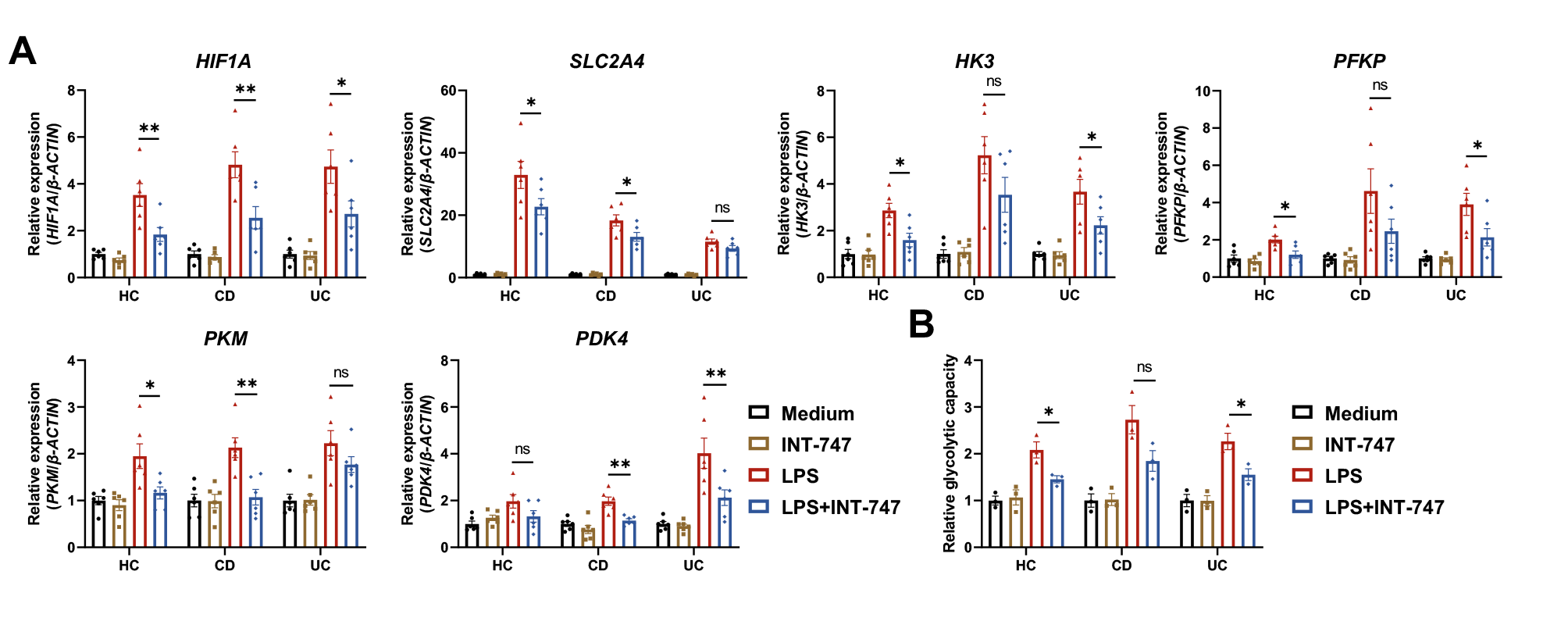


**Supplementary Figure 9.** **FXR signaling downregulates glycolysis-related genes in neutrophils.** (**A**) PB neutrophils (3 × 10^6^/mL) isolated from healthy donors (HC, n=6), patients with active CD (n=6), and patients with active UC (n=6) were stimulated with LPS (300 ng/mL) in the presence or absence of INT-747 (40 μM) for 3 hours. The expression levels of glycolysis-related genes were measured by qRT-PCR. (**B**) Bar chart of the relative glycolytic capacity described in Figure 6G. Data were expressed as mean ± SEM. Tukey’s test was used for statistical analysis. **p*<0.05; ***p*<0.01; ****p*<0.001; *****p*<0.0001; ns, not significant.

**Supplementary tables**

**Supplementary Table 1.** **Reagents used in this study**

| **Reagents** | **Sources** | **Identifiers** |
| --- | --- | --- |
| **Chemical Reagents** | | |
| Dextran sulfate sodium salt | MP Biomedicals | 9011-18-1 |
| INT-747 | MedChemExpress | HY-12222 |
| Rapamycin | MedChemExpress | HY-10219 |
| 2-Deoxy-D-glucose (2-DG) | MedChemExpress | HY-13966 |
| Lipopolysaccharide (LPS) | Sigma-Aldrich | 297-473-0 |
| Phorbol 12-myristate 13-acetate (PMA) | Sigma-Aldrich | 16561-29-8 |
| *N*-formy-lmethionyl-leucyl-phenyl-alanine (fMLP) | Sigma-Aldrich | 59880-97-6 |
| Recombinant Human IL-8 Protein | R&D systems | 208-IL-010 |
| Flagellin | InvivoGen | Tlrl-stfla |
| Peptidoglycan (PGN) | Sigma-Aldrich | 69554 |
| Pam3CSK4 | APExBIO | B5662 |
| Fibroblast-stimulating lipopeptide-1 (FSL-1) | Abcam | Ab144863 |
| CM-H_2_DCFDA | Invitrogen | C6827 |
| 2-NBDG | Invitrogen | N13195 |
| DAPI | Invitrogen | R37606 |
| Hoechst 33342 | MedChemExpress | HY15559 |
| Crystal violet | Sangon Biotech | A600331 |
| Ficoll-Paque^TM^ PLUS density gradient media | Cytiva | 17144002 |
| BD Pharm Lyse^TM^ Lysing Buffer | BD Biosciences | 555899 |
| **Reagents** | **Sources** | **Identifiers** |
| eBioscience^TM^ FOXP3/Transcription Factor Staining Buffer Set | Invitrogen | 00-5523-00 |
| IC Fixation Buffer | Invitrogen | 00-8222-49 |
| **Immunological Microbeads** |  |  |
| Anti-Human CD14 Magnetic Particles | BD Biosciences | 557769 |
| Anti-Human CD4 Magnetic Particles | BD Biosciences | 557767 |
| Anti-Human CD8 Magnetic Particles | BD Biosciences | 557766 |
| Anti-Human CD19 Magnetic Particles | BD Biosciences | 551520 |
| Anti-Human CD56 Magnetic Particles | BD Biosciences | 557775 |
| **Antibodies** |  |  |
| FITC anti-mouse CD11b Antibody | BioLegend | 101206 |
| APC anti-mouse Ly-6G Antibody | BioLegend | 127614 |
| PE anti-mouse F4/80 Antibody | BioLegend | 123110 |
| APC anti-mouse CD3 Antibody | BioLegend | 100236 |
| PE anti-mouse CD4 Antibody | BioLegend | 100408 |
| Anti-Bile Acid Receptor NR1H4 Antibody | Abcam | Ab187735 |
| Anti-Histone H3 (citrulline R2+R8+R17) Antibody | Abcam | Ab5103 |
| Anti-Myeloperoxidase Antibody (rabbit anti-mouse/human) | Abcam | Ab208670 |
| Anti-Myeloperoxidase Antibody (mouse anti-human) | Abmart | MU135210 |
| Anti-Myeloperoxidase Antibody (mouse anti-mouse) | HUABIO | EM1901-19 |
| Goat anti-Mouse IgG (H+L) Highly Cross-Adsorbed Secondary Antibody, Alexa Fluor™ Plus 488 | Invitrogen | A32723TR |
| Goat anti-Rabbit IgG (H+L) Highly Cross-Adsorbed Secondary Antibody, Alexa Fluor™ 594 | Invitrogen | A-11037 |
| **Reagents** | **Sources** | **Identifiers** |
| Phospho-S6 Ribosomal Protein (Ser240/244) (D68F8) XP^®^ Rabbit mAb | CST | 14236 |
| Phospho-4E-BP1 (Thr37/46) (236B4) Rabbit mAb | CST | 2846 |
| Phospho-Akt (Ser473) (D9E) XP^®^ Rabbit mAb | CST | 5135 |
| Phospho-mTOR (Ser2448) Monoclonal Antibody (MRRBY) | Invitrogen | 25-9718 |

**Supplementary Table 2.** **Characteristics of IBD patients and healthy donors**

|  | HC  (n=99) | A-CD  (n=81) | R-CD  (n=36) | A-UC  (n=81) | R-UC  (n=36) |
| --- | --- | --- | --- | --- | --- |
| Age, y (mean±SD) | 29.3±5.4 | 35.8±13.2 | 34.3±13.0 | 34.5±15.0 | 30.9±14.5 |
| Gender, n (%) |  |  |  |  |  |
| Female | 51 (51.5) | 25 (30.9) | 8 (22.2) | 36 (44.4) | 18 (50.0) |
| Male | 48 (48.5) | 56 (69.1) | 28 (77.8) | 45 (55.6) | 18 (50.0) |
| Disease duration, m (mean±SD) |  | 39.1±37.9 | 46.0±36.3 | 35.1±32.1 | 26.7±29.5 |
| Lesion location (UC)*, n (%) |  |  |  |  |  |
| E1 |  |  |  | 16 (19.8) | 17 (47.2) |
| E2 |  |  |  | 45 (55.6) | 10 (27.8) |
| E3 |  |  |  | 20 (24.7) | 9 (25.0) |
| Lesion location (CD)*, n (%) |  |  |  |  |  |
| L1 |  | 19 (23.5) | 9 (25.0) |  |  |
| L2 |  | 19 (23.5) | 8 (22.2) |  |  |
| L3 |  | 43 (53.1) | 19 (52.8) |  |  |
| Mayo index (mean±SD) |  |  |  | 7.7±2.8 | 1.2±0.6 |
| CDAI (mean±SD) |  | 310.1±95.2 | 104.0±23.8 |  |  |
| Current therapy, n (%) |  |  |  |  |  |
| 5-ASA |  | 17 (21.0) | 14 (38.9) | 18 (22.2) | 14 (38.9) |
| Glucocorticoids |  | 10 (12.3) | 9 (25.0) | 9 (11.1) | 9 (25.0) |
| Immunosuppressants |  | 6 (7.4) | 4 (11.1) | 7 (8.6) | 4 (11.1) |
| Infliximab |  | 2 (2.5) | 13 (36.1) | 3 (3.7) | 12 (33.3) |

*According to the Montreal classification

**Supplementary Table 3.** **Primers used for qRT-PCR analysis**

| **Gene** | **Species** | **Forward Sequence (5’ to 3’)** | **Reverse Sequence (5’ to 3’)** |
| --- | --- | --- | --- |
| *Gapdh* | Mouse | CATCACTGCCACCCAGAAGACTG | ATGCCAGTGAGCTTCCCGTTCAG |
| *Actb* | Mouse | CATTGCTGACAGGATGCAGAAGG | TGCTGGAAGGTGGACAGTGAGG |
| *Tnf* | Mouse | GGTGCCTATGTCTCAGCCTCTT | GCCATAGAACTGATGAGAGGGAG |
| *Il6* | Mouse | TACCACTTCACAAGTCGGAGGC | CTGCAAGTGCATCATCGTTGTTC |
| *S100a8* | Mouse | CAAGGAAATCACCATGCCCTCTA | ACCATCGCAAGGAACTCCTCGA |
| *S100a9* | Mouse | TGGTGGAAGCACAGTTGGCAAC | CAGCATCATACACTCCTCAAAGC |
| *Lcn2* | Mouse | ATGTCACCTCCATCCTGGTCAG | GCCACTTGCACATTGTAGCTCTG |
| *Cxcl1* | Mouse | TCCAGAGCTTGAAGGTGTTGCC | AACCAAGGGAGCTTCAGGGTCA |
| *Ifng* | Mouse | CAGCAACAGCAAGGCGAAAAAGG | TTTCCGCTTCCTGAGGCTGGAT |
| *Il17a* | Mouse | CAGACTACCTCAACCGTTCCAC | TCCAGCTTTCCCTCCGCATTGA |
| *Il1b* | Mouse | TGGACCTTCCAGGATGAGGACA | GTTCATCTCGGAGCCTGTAGTG |
| *Hif1a* | Mouse | CCTGCACTGAATCAAGAGGTTGC | CCATCAGAAGGACTTGCTGGCT |
| *Slc2a4* | Mouse | GGTGTGGTCAATACGGTCTTCAC | AGCAGAGCCACGGTCATCAAGA |
| *Hk3* | Mouse | CTGAGTCAAGGCTGTATCCTCC | TGCACCAGTTCAGCATCTGAGG |
| *Pfkp* | Mouse | AAGAGGAAACCAAGCAGTGCGC | TTCCTCGGAGTTTCACGGCTTC |
| *Pkm* | Mouse | CAGAGAAGGTCTTCCTGGCTCA | GCCACATCACTGCCTTCAGCAC |
| *Pdk4* | Mouse | GTCGAGCATCAAGAAAACCGTCC | GCGGTCAGTAATCCTCAGAGGA |
| *GAPDH* | Human | GTCTCCTCTGACTTCAACAGCG | ACCACCCTGTTGCTGTAGCCAA |
| *ACTB* | Human | CACCATTGGCAATGAGCGGTTC | AGGTCTTTGCGGATGTCCACGT |
| *NR1H4* | Human | ACTTCCGTCTGGGCATTCTGAC | GCTGTAAGCAGAGCATACTCCTC |
| *GPBAR1* | Human | GCTGCTTCTTCCTGAGCCTACT | TTGGGAGCCAAGTAGACGAGGA |
| *VDR* | Human | CGCATCATTGCCATACTGCTGG | CCACCATCATTCACACGAACTGG |
| *S1PR2* | Human | TGGAAACGCAGGAGACGACCTC | CGAGTGGAACTTGCTGTTTCGG |
| *TNF* | Human | CTCTTCTGCCTGCTGCACTTTG | ATGGGCTACAGGCTTGTCACTC |
| *IL1B* | Human | CCACAGACCTTCCAGGAGAATG | GTGCAGTTCAGTGATCGTACAGG |
| *IL6* | Human | AGACAGCCACTCACCTCTTCAG | TTCTGCCAGTGCCTCTTTGCTG |
| *S100A8* | Human | ATGCCGTCTACAGGGATGACCT | AGAATGAGGAACTCCTGGAAGTTA |
| *S100A9* | Human | GCACCCAGACACCCTGAACCA | TGTGTCCAGGTCCTCCATGATG |
| *LCN2* | Human | GTGAGCACCAACTACAACCAGC | GTTCCGAAGTCAGCTCCTTGGT |
| *IL8* | Human | GAGAGTGATTGAGAGTGGACCAC | CACAACCCTCTGCACCCAGTTT |
| *HIF1A* | Human | TATGAGCCAGAAGAACTTTTAGGC | CACCTCTTTTGGCAAGCATCCTG |
| *SLC2A4* | Human | CCATCCTGATGACTGTGGCTCT | GCCACGATGAACCAAGGAATGG |
| *HK3* | Human | CATCGTGGACTTCCAGCAGAAG | CTTGGTCCAGTTCAGGAGGATG |
| *PFKP* | Human | AGGCAGTCATCGCCTTGCTAGA | ATCGCCTTCTGCACATCCTGAG |
| *PKM* | Human | ATGGCTGACACATTCCTGGAGC | CCTTCAACGTCTCCACTGATCG |
| *PDK4* | Human | AGGTGGAGCATTTCTCGCGCTA | GAATGTTGGCGAGTCTCACAGG |
